# The Role of Extracellular Vesicles in Transient Ischaemic Attacks and Ischaemic Stroke: A Systematic Review

**DOI:** 10.1002/jex2.70124

**Published:** 2026-03-13

**Authors:** Rebecca Marie Raven, Philip Eurig James, Keith Morris, Jessica Olivia Williams

**Affiliations:** ^1^ CURIAD, Centre of Cardiovascular Research Innovation and Development Cardiff Metropolitan University Cardiff UK

**Keywords:** biomarker, extracellular vesicles, ischaemic stroke, transient ischaemic attack

## Abstract

Stroke is the second leading cause of death and a major contributor of long‐term disability globally. Ischaemic stroke (IS) is the most common type of stroke; characterised by a blood clot that causes oxygen deprivation in the brain. Survivors often face long‐lasting disabilities, imposing significant social and economic burden. Up to a quarter of IS are preceded by a transient ischaemic attack (TIA), where temporary symptoms occur due to a short‐term interruption in blood flow. Currently, there is no reliable method to identify which TIA patients are at greatest risk of stroke. Early identification of circulating biomarkers in this cohort could inform clinical follow‐up and help prevent future strokes. Extracellular vesicles (EVs) are membrane bound nanoparticles, released by all cell types playing key roles in cell‐to‐cell communication. EVs have recently emerged as effective biomarkers in disease diagnostics, yet their involvement in IS and TIA remains poorly understood. To explore this, a systematic review was undertaken following the Preferred Reporting Items for Systematic Reviews and Meta‐Analyses (PRISMA) Guidelines. A comprehensive search of PubMed, Scopus and Web of Science up to May 2025 yielded 1206 articles, of which 31 met the inclusion criteria and were analysed. This review highlights the necessity for standardised methodologies, particularly in EV isolation and characterisation, to allow data comparability and clarity of the role of EVs in the pathophysiology of IS and TIA.

## Graphical abstract

Both transient ischaemic attack (TIA) and ischaemic stroke lead to an increase in individual cellular‐derived populations of extracellular vesicles (EVs), including endothelial, platelet and leukocyte derived. These EVs have downstream roles in the pathophysiological events associated with TIA and stroke, including dysregulated inflammation and coagulation. EVs expressing cellular markers have been posed as a suitable prognostic biomarker in these cohorts

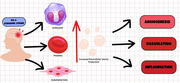

## 1 Introduction

Stroke is the second leading cause of death and disability worldwide with the number of strokes predicted to rise by 60% between 2015 and 2035 (Dohle et al. 2025). Ischaemic stroke (IS) accounts for over 85% of all strokes and is the result of thrombus formation within a cerebral blood vessel resulting in disruption to the supply of oxygen rich blood within the brain (Alkarithi et al. 2021). Up to 25% of ISs are preceded by a ‘Transient Ischaemic Attack’ (TIA), commonly referred to as a ‘mini‐stroke’, whereby a temporary interruption of blood supply to the brain occurs, with symptoms typically lasting no longer than 4 h. Specifically, TIA sufferers are at a considerably higher risk of stroke within the first 24 h following a TIA, with risk persisting up to 5 years later (Vinding et al. 2023). For healthcare professionals, identifying those TIA patients who are most likely to suffer a disabling stroke is a crucial step in reducing stroke burden. Understanding the biological processes that infer risk following a TIA, along with identifying potential biomarkers would allow tailored treatment intervention to those with highest risk.

Extracellular vesicles (EVs) are sub‐micron, membrane contained vesicles released by cells into the circulation. EVs are important intercellular communication mediators making them a major research topic in diagnostic biomedicine and the pathophysiology of disease. EV subsets (exosomes, microvesicles and apoptotic bodies) are well characterised and broadly split according to their origin and mechanisms of production. Traceable protein markers are expressed on the EV surface that are representative of the cell of origin and reflect its physiological/pathophysiological state (Novikova et al. 2021; Liu et al. 2023; Rontogianni et al. 2019). This allows EV subtype identification in mixed plasma samples as we and others have previously shown (Connolly et al. 2018; Holcar et al. 2025; Vagner et al. 2018). In recent years, plasma‐derived EVs have been proposed as potential diagnostic and prognostic biomarkers. Studies have identified EVs are linked to stroke pathology, with clear associations to stroke severity and infarct size (Escudero‐Guevara et al. 2025). It is evident that EV research has suffered from a lack of standardisation in both methodology and terminology. This situation has somewhat been improved with the introduction of the International Society of EV (ISEV) producing guidelines for EV research (Welsh et al. 2024). Although several literature reviews have been published reporting on EVs in stroke, none have included a full systematic review of the literature on EVs in IS and TIA, few have applied a structured assessment of quality or standardisation making comparison difficult.

The objective of this systematic review was to critically evaluate the evidence for a role of EV in the pathophysiology of TIA and IS, with a specific focus on the use and reporting of standardised EV isolation and characterisation methodologies, through applying a bespoke rating scale to assess validity and comparability of reported findings.

## 2 Methods

### 2.1 Protocol and Registration

This comprehensive systematic review was conducted according to the Preferred Reporting Items for Systematic Reviews and Meta‐Analyses (PRISMA) guidelines (Page et al. 2021). The protocol was registered on PROSPERO (CRD42023456719) and obtained ethical approval under Cardiff Metropolitan University Ethics Framework (PGT‐9454).

### 2.2 Literature Search

The databases PubMed, Scopus and Web of Science were searched from date of inception for results up 31 May 2025. The search terms were adapted according to the bibliographic databases and included (EVs or exosomes or microvesicles or microparticles) and (IS or TIA). Searches were specific to only include publications written in English, and conference abstracts were excluded from the search. The full search syntax strategy is detailed in Supporting Information (S1).

### 2.3 Study Quality Assessment

To assess the quality of the publications reporting on EVs in TIAs and IS, the studies were screened, by two independent assessors, for components concerning the clinical cohorts (TIA and IS), EV isolation and characterisation. Only human studies were included to ensure clinical relevance and methodological comparability. TIA has no directly comparable animal correlate due to the heterogeneity of the disease. EV composition and protein expression are also known to differ between biological species (Wang et al. 2020).

To support a structured comparison between the studies included, a bespoke methodological appraisal score was developed. The score was developed to capture key aspects of the study design, diagnostic ascertainment, EV isolation and characterisation methodologies that are relevant to the review outcomes. The appraisal score reflects reported experimental practices that align with MISEV criteria, independently of citation of the guidelines, with an additional point awarded where studies explicitly referenced MISEV. Overall, the score reflects study size and comparators, diagnostic confirmation and completeness of EV methodology.

Due to small sample sizes having potentially increased inherent variability due to EV analysis, evaluation on the study population was based on size (score: *n*/3) and number of control groups (score *n*/2). Population size was scored using the following scale: small study size (*n* ≤ 20) = 1, large size (*n* ≥ 20) = 2, large size and control groups = 3. The studies were then scored for inclusion of control groups (*n*/2), where no control group = 0, one control group = 1 and two control groups = 2. Studies were assessed for the method of diagnosis of TIA or IS (score *n*/2), where not stated = 0, diagnosis by symptoms = 1 and by MRI or CT = 2. EV research is a developing field, and criteria need to be set regarding the experimental approach and techniques used. EV isolation and characterisation techniques were scored out of (*n*/2) and (*n*/3), respectively. No mention of EV isolation was scored 0, at least three differential centrifugation steps = 1 and additional size exclusion chromatography (SEC) or ultracentrifugation = 2. An extra point was awarded to studies if minimal information for the studies of EVs (MISEVs) criteria were mentioned. The methods used for characterisation of EV was ranked (*n*/3), whereby no analysis = 0, one method = 1, mention of two out of three characterisation methods outlined in rank 3 = 2, mentions all parameters of EV concentration and size, surface proteins and content = 3. Together the total was 13 marks for all components added. This allowed for study ranking to be calculated (score = (1–13) × number of marks given for paper), from a score of 1.0, where 1.0 was the highest ranking. An overall credibility score of 0 shows that the study does not conform to any standards and a score of 1 means the study conforms to all standards set.

## 3 Results

### 3.1 Literature Review

**FIGURE 1:**
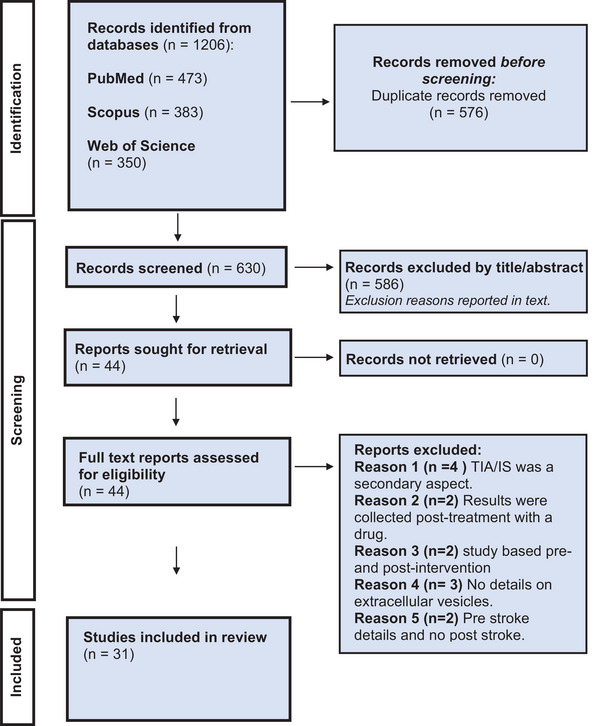
Preferred Reporting Items for Systematic Reviews and Meta‐Analysis (PRISMA) 2020 guidelines flow diagram representing the research and selection process for this review.

**FIGURE 2:**
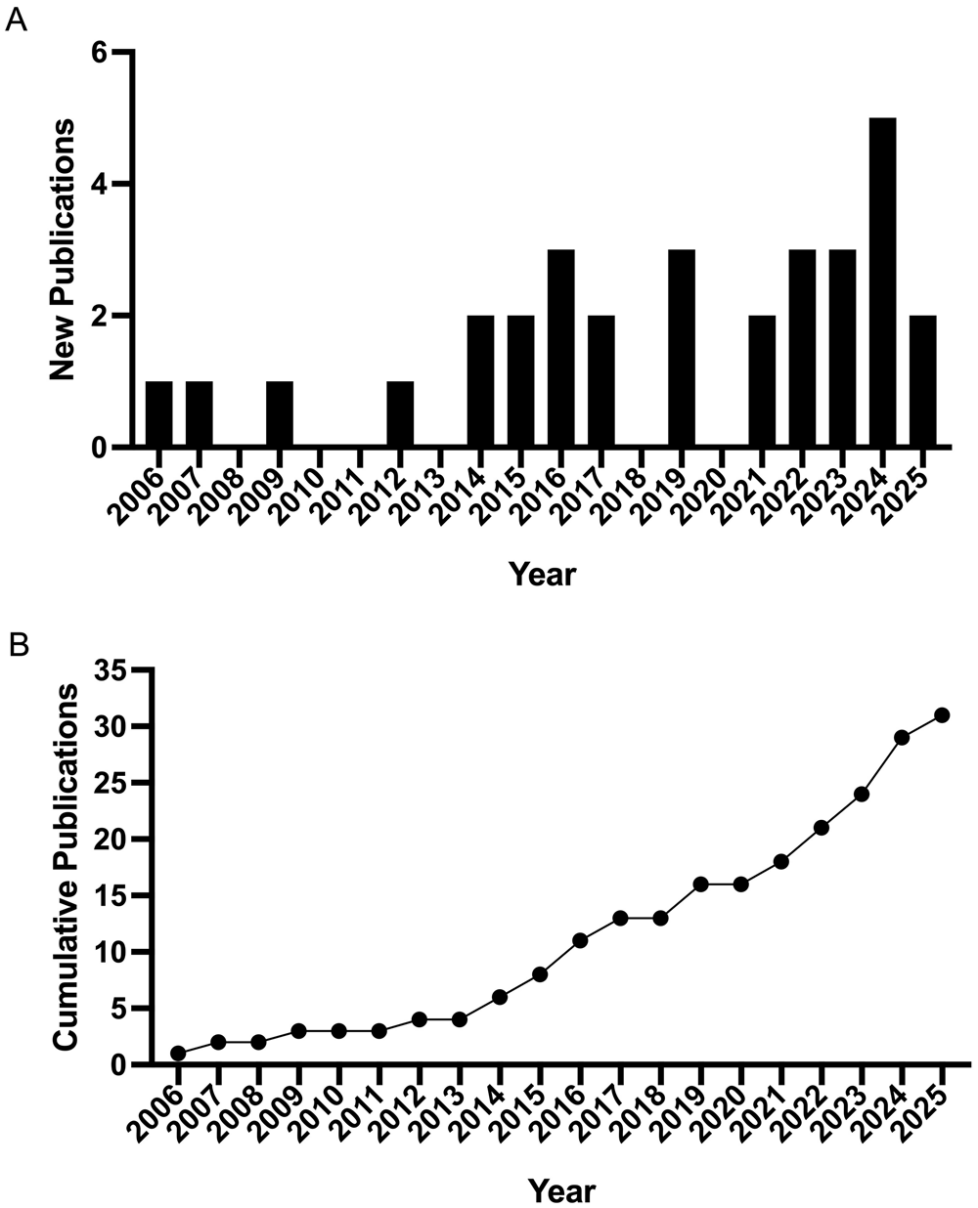
Results of the literature search. The graphs show the (A) number of publications per year and (B) cumulative frequency of total publications per year from database inception for our matched search terms.

In total, 1206 papers were retrieved using the search strategy outlined. A total of 473 from PubMed, 383 from Scopus and 350 from Web of Science. Duplicates were removed (*n* = 576) leaving 630 papers whose titles and abstracts were screened. A total of 586 articles were excluded for various reasons: the study does not include humans, or where animal (*n* = 240) or cell‐based (n = 87) stroke models, studies focussing on miRNA (*n* = 58), review articles (*n* = 53), case studies (*n* = 8), not EV based (*n* = 29), patients were under 18‐year‐old (*n* = 3), therapeutic interventions (*n* = 11) they were not TIA/IS specific (*n* = 55) or other (*n* = 44). The remaining 44 articles were retrieved for full paper review, of which 31 fulfilled the eligibility criteria and were included in the present review. Exclusion reasons for the articles are listed in Figure 1, which outlines the review process in a detailed flow diagram. The first study was published in 2006 with the number of publications increasing in more recent years and peaking with a total of 31 publications by 31 May 2025 (Figure 2A, B). Table 1 summarises the articles included and analysed in the review, and further details can be found in Supporting Information (Tables S1–S3).

**TABLE 1:** Summary of studies on EVs in transient ischaemic attacks and ischaemic stroke.

Reference	Characteristics; age, gender, total participants, diagnosis, treatment, vascular risk factors, clinical severity	Aims of study/outcomes measured	Main findings
Ortega et al. (2021)	Patient Group 1 CSC Mean age = 71 56.4% F 43.6% M *N* = 55 Patient Group 2 SC Mean age = 62 30.8%F 69.2% M *N* = 26 Healthy control group Mean age = 61 68.2% F 31.8% M *N* = 22	To analyse whether EV levels, proteins and microRNAs could be modified based on the differing lesion topography in patients with SC and CSC ischaemic stroke.	EV concentration: No significant differences between SC, CSC or HC. No correlation between EV concentration the infarct volume. Sampling time point: 1. 1 day 2. 7 days
	Treatment status: data not shown		EV size: Data were not reported.
			EV content: The CSC group showed specific expression of complement C1q A chain (C1QA) and alpha‐2‐macroglobulin (A2M) EV. CSC patients showed significantly higher fibrinogen C (FGC), C4b‐binding protein alpha chain precursor (C4BPA), fibronectin‐1 (FN1) and Von Willebrand factor (VWF) in EV compared to HCs (*p* = 0.014, *p* = 0.003, *p* = 0.002 and *p* = 0.043). The SC group showed specific expression of Annexin‐2 (ANXA2) EV. The study isolated neuronal‐derived EVs (NDEs) (L1CAM+) and identified 1279 proteins inside. The specific proteins of the CSC (C1QA, caspase 14 [Casp14]) and SC (ANXA2) were of the most abundant proteins. Apolipoprotein B‐100 (APOB), complement Component 3 (C3), FN1, VWF and FCG are abundant in NDEs. EVs in TIA or stroke: IS
	Risk factors (CSC vs. SC group); 1. Hypertension; 63% vs. 73% 2. Diabetes mellitus; 18% vs. 19% 3. Dyslipidaemia; 45% vs. 42% 4. Smoker; 16% vs. 35% 5. Coronary artery disease; 10% vs. 0% 6. Atrial fibrillation; 22% vs. 7% Clinical severity; NIHSS CSC = 9 NIHSS SC = 3		Main finding: An integrated analysis of the proteome and transcriptome revealed differences in circulating EV proteins depending on ischaemic stroke topography.
Yao et al. (2017)	Patient group IS Mean age = 65 42.1%F, 57.9% M *N* = 79 Control Group 1 Risk factor matched Mean age = 63 42.9% F, 57.1% M *N* = 35 Control Group 2 Healthy control 63 46.9% F, 53.1% M *N* = 32	To determine the role of phosphatidylserine (PS)‐mediated procoagulant activity (PCA) in stroke. To ascertain this role, the early dynamic evolution of PS exposure on blood cells and released EV and the corresponding PCA were evaluated in patients with IS.	EV concentration: Total PS + EV were increased in AIS compared with risk factor matched (RF) (*p* = 0.019) and HC (*p* < 0.001).
	Treatment status: 1. Pre‐Sample; 3 thrombectomy and 7 anti‐coagulated 2. Post‐Sample 1; all anti‐coagulated		Sampling time point: 1. <1 day 2. 1 day 3. 3 days 4. 7 days EV size: Data were not reported.
	Risk factors (stroke vs. matched control) 1. Hypertension; 45% vs. 48% 2. Diabetes mellitus; 29% vs. 31% 3. Dyslipidaemia; 21% vs. 25% 4. Smoker; 24% vs. 28% 5. Coronary artery disease; 21% vs. 23% 6. Atrial fibrillation; 24% vs. 25%		
	Clinical severity; NIHSS = 6		EV content: Flow cytometry analyses revealed that initial levels of PS exposure on erythrocyte, platelet and leukocyte were 2.40‐, 1.36‐ and 1.38‐ fold higher, respectively, in AIS than the risk factor‐matched controls. Platelets PS + EVs increased in AIS compared to controls (*p* < 0.01). EVs in TIA and stroke: IS.
			Main finding: The thrombotic susceptibility of IS patients can be partly ascribed to PS exposure on blood cells and the release of EV. The study identified PS exposure as a potentially novel therapeutic target in the treatment of IS.
Datta et al. (2014)^a^	Patient group Lacunar infarction (LACI) Mean age = 61 45% F, 55% M *N* = 45 patients Control group Mean age = 56 74% F, 26% M *N* = 17	To determine if LACI patients have poor mid‐ to long‐term prognosis due to the recurrence of vascular events or a decline in cognitive function in association to EVs.	EV Concentration: Data not reported. Sampling time point: 1. Within 180 days
	Treatment status: 1. Aspirin 2. Aspirin + dipyridamole		EV size: Data not reported. EV content: Proteins related to ‘enzyme inhibitor activity’—e.g., complement proteins; C5 and C3, vitamin K‐dependent protein S and inter‐alpha (globulin) inhibitor H4 (ITIH4) are upregulated in the LACI group with better outcome and downregulated in the group with adverse outcomes. Proteins related to ‘integrin cell surface interactions’ (e.g., ITGA2B, TLN1, FGB and FGA) were downregulated in LACI patients with no adverse outcome while upregulated in both groups with adverse outcome. The brain‐specific myelin basic protein (mbp) was found in higher concentration compared to controls. Downregulation of albumin in EV rich plasma was associated with adverse outcome among LACI patients.
	Risk factors (no future event vs. recurrent vascular events) 1. Hypertension; 58% vs. 82% 2. Diabetes mellitus; 37% vs. 18% 3. Dyslipidaemia; 42% vs. 36% 4. Smoker; 26% vs. 55% 5. Coronary artery disease; data not reported 6. Atrial fibrillation; data not reported Clinical severity; Modified Rankin score ≤ 3		EVs in TIA and stroke: LACI IS (25% of all IS). Main finding: The study identified up‐regulation of brain‐specific proteins including myelin basic protein, proteins of coagulation cascade (e.g., fibrinogen alpha chain, fibrinogen beta chain) and focal adhesion (e.g., integrin alpha‐IIb, talin‐1 and filamin‐A), while albumin was downregulated in EV rich plasma of both groups of patients with adverse outcome compared to controls.
Cappelletti et al. (2022)	Patient group Ischaemic stroke Mean age = 70 50% F, 50% M *N* = 30 Control Group 1 Age matched (HC) Mean age = 72 50% F, 50% M *N* = 30 Control Group 2 Young controls (Y‐HC) Mean age = 33 60% F, 40% M *N* = 15	To analyse the expression levels of two synaptic proteins Syntaxin (STX)‐1a and Synaptosomal associated protein, 25 kDa (SNAP‐25), in peripheral blood mononuclear cell (PBMC), serum and in neuronal derived EV (NDEs).	EV concentration: Data were not reported.
			Sampling time point: Data were not reported.
	Treatment status; 59% Anti‐platelet, 41% anti‐platelet and anti‐coagulant		EV size: Data were not reported.
	Risk factors; data not shown		EV content: Total EVs STX‐1 was not quantifiable when NDEs were depleted from EV fraction. STX‐1 was significantly increased in NDEs derived from IS patients compared to HCs (*p* < 0.0001). SNAP‐25 expression on EVs was not significantly different between groups. In NDEs SNAP‐25 expression is statistically higher in NDEs in IS to HC (*p* = 0.02).
	Clinical severity; NIHSS = 4.9		EV in stroke or TIA: IS
			Main finding: STX‐1a and SNAP‐25 levels were significantly enriched in NDEs purified from the serum of IS patients compared with HC.
Rosinska et al. (2019)	Patient Group 1 Large artery atherosclerosis (LAA) Mean age = 66 (63–77) 22% F, 78% M *N* = 40 Patient Group 2 Small arteries occlusion (SAO) Mean age = 66 (60–74) 62% F, 38% M *N* = 32 RF matched control group Mean age = 65 (60–72) 51% F, 49% M *N* = 69	Association of platelet‐derived EV and their phenotypes with carotid atherosclerosis and recurrent vascular events in patients after ischemic stroke.	EV concentration: Data not reported.
			Sampling time point: 1. Between 90 and 365 days
	Treatment status; Acetylsalicylic acid		EV size: Data not reported.
			EV content: Total pMVs are defined as CD61+ no difference in total between groups. CD62p/CD61+ EV/uL and PAC‐1/CD61+ EV/uL (AB against GP11b [platelet receptor]) higher in stroke patients with carotid atherosclerotic plaques and increased carotid IMT (*p* < 0.05).
	Risk factors (LAA vs. SAO vs. control) 1. Hypertension; 95% vs. 94% vs. 94% 2. Diabetes mellitus; 38% vs. 31% vs. 31% 3. Dyslipidaemia; 95% vs. 88% vs. 90% 4. Smoker; 38% vs. 31% vs. 36% 5. Coronary artery disease; data not reported 6. Atrial fibrillation; data not reported		EV in stroke or TIA: IS, large area atherosclerosis vs. small artery occlusion vs. controls.
	Clinical severity; Data not shown		Main finding: PAC1 and CD62p+ CD61+EV are associated with carotid and intima thickness in stroke patients.
Chiva‐Blanch et al. (2016)	Patient group IS Mean age = 70 34% F, 66% M *N* = 44 Control group Mean age = 73 66% F, 34% M *N* = 44	Aimed to determine EV shedding from different cells at the onset of stroke and at 7 and 90 days and compare it to subjects at high risk but without cardiovascular disease. Aimed to analyse the association between EV and cerebral infarction size, aetiology and level of chronic disability in patients with IS.	EV concentration: Total EV was determined using annexin V positivity. Significantly increased in stroke vs. control; 343.10 vs. 693.56 (*p* < 0.001).
			Sampling time point: 1. 0 days 2. 7 days 3. 90 days
	Treatment status; 1. Thrombolysis 23% stroke group		EV size: Data not reported.
	Risk factors (stroke vs. control) 1. Hypertension; 64% vs. 50% 2. Diabetes mellitus; 30% vs. 14% 3. Dyslipidaemia; data not shown 4. Smoker; 20% vs. 18% 5. Coronary artery disease; data not shown 6. Atrial fibrillation; data not shown		EV content: EV of the following subtypes were. Increased in IS patients *p* < 0.001 for all unless stated; Neural progenitor cells (CD34 and CD56 and AV+), platelet (CD61+, CD142+), activated platelet (CD62p+), endothelial (CD146+, CD62e+), red blood cell (CD235a+), leucocyte (CD45+, CD62+, CD11a+), lymphocyte (CD3+), monocyte (CD144+, CD11a+ (*p* = 0.003), CD142+ (*p* = 0.005).
	Clinical severity; data not shown		EV in stroke or TIA: IS
			Main Finding: Stroke increases blood and vascular compartment cell and neural precursor cell EV shedding, an effect that is chronically maintained up to 90 days after the IS.
Eyileten et al. (2022)	Patient group IS Mean age = 66 46.4% F, 53.6% M *N* = 28 CAD control group Mean age = 65 40% F, 60% M *N* = 35	Aimed to assess circulating EVs from platelets, leukocytes and endothelial cells to analyse their diagnostic and predictive utility in patients with acute IS. The concentrations of EVs from platelets, leukocytes and endothelial cells were evaluated in patients with IS.	EV Concentration: Data not reported.
			Sampling time point: 1. 1 day 2. 7 days
	Treatment status; data not shown		EV size: Data not reported.
	Risk factors (stroke vs. control) 7. Hypertension; 64% vs. 63% 8. Diabetes mellitus; 17% vs. 20% 9. Dyslipidaemia; data not shown 10. Smoker; 39% vs. 23% 11. Coronary artery disease; 100% vs. 28% 12. Atrial fibrillation; 11% vs. 0%		EV content: HPR = high platelet reactivity Patients with high platelet reactivity had significantly elevated platelets EVs (CD62+) *p* = 0.012 and leukocyte EVs (CD45+) *p* = 0.002. IS patients had significantly higher platelet‐EVs (CD61+) concentration both at Day‐1 and ‐7 post‐stroke compared to control patients (*p* = 0.001, *p* = 0.030, respectively). Leukocyte‐EVs (CD45+) were significantly higher in IS patients at Day‐1 compared to the control group (*p* = 0.005). No significant differences were observed for leukocyte‐EVs concentration between control patients and patients at Day‐7 post‐stroke (*p* = 0.104).
	Clinical severity; 1. NIHSS (1–4) 54% 2. NIHSS (5–15) 46%		EV in stroke and TIA: IS
			Main finding: Increased concentrations of EVs from platelets, leukocytes and endothelial cells in patients with IS in the first 24 h after ischaemic episode.
Maciejewska‐Renkowska et al. (2024)	Patient group IS Mean age = 69 48% F, 52% M *N* = 168 Control Group 1 Healthy controls (HC) Mean age = 44 43% F, 57% M *N* = 21 Control Group 2 Control group with vascular disease risk factors (DC) Mean age = 67 42% F, 58% M *N* = 63	To determine levels of circulating platelet EVs and characterise their phenotype in IS at different timepoints (D1, D3 = acute; D10 = subacute; convalescent = D90) compared to a healthy control (HC) group and a disease‐matched group (DC).	EV concentration: Data not reported.
			Sampling time point: 1. 1 day 2. 3 days 3. 10 days 4. 90 days
	Treatment status; 1. Thromboylsis 32% stroke group		EV size: Data not reported.
	Risk factors (stroke vs. DC) 13. Hypertension; 76% vs. 75% 14. Diabetes mellitus; 23% vs. 19% 15. Dyslipidaemia; 33% vs. 35% 16. Smoker; 24% vs. 22% 17. Coronary artery disease; 32% vs. 41% 18. Atrial fibrillation; 34% vs. 37%		EV content: Concentration of platelet (pEV) EV were significantly higher at all phases of stroke (D1‐3, D10, D90) compared to HC (*p* < 0.05 for all). The concentration of pEVs on Days 1 and 3 did not differ in comparison to DC group but was significantly lower at Day 10 (*p* < 0.002) and Day 90 (*p* < 0.006). PS + pEVs were higher after stroke vs. controls (*p* < 0.001). The concentration of pEVs with expression of surface markers (CD62P, CD40L and PECAM‐1) was higher on Days 1 and 3 after stroke compared to both control groups. The concentrations remained higher at Days 10 and 90 for IS compared to HCs (*p* < 0.05).
	Clinical severity; NIHSS = 4		EV in stroke or TIA: IS
			Main finding: IS affected the phenotype of pEVs in comparison to HC and DCs but a limited effect on concentrations of pEV was observed.
Reymond et al. (2025)	Patient Group 1 Ischaemic stroke *N* = 20 2 Subgroups LVO strict Mean age = 80 60% F, 40% M *N* = 10 Non‐LVO IS Mean age = 73.5 50% F, 50% M *N* = 10 Patient Group 2 Haemorrhagic Mean age = 77.5 30% F, 70% M *N* = 10 Patient Group 3 TIA Mean age = 84.5 50% F, 50% M *N* = 10 Patient Group 4 Mimics Mean age = 76.5 70% F, 30% M Control group Mean age = 73.5 50% F, 50% M *N* = 10	To identify the protein content of plasma derived EVs in a cohort composed of patients with various stroke subtypes (haemorrhagic, ischaemic ± LVO, TIA, Mimic) and compare to a healthy control cohort.	EV concentration: No difference in particle concentration was identified between stroke subtypes and controls (*p* > 0.05).
			Sampling time point: 1. <1 day
	Treatment status: Data not reported		EV size: No difference in particle size was identified between stroke subtypes and controls (*p* > 0.05).
			EV content: Quantification of over 600 proteins. The proteins separating the healthy controls were mainly related to platelet formation. Large overall protein characterisation but mainly cluster analysed for results and needs to be further researched with bigger groups.
	Risk factors (Group 1 vs. 2 vs. 3 vs. 4 vs. 5) 19. Hypertension; 60% vs. 70% vs. 70% vs. 70% vs. 100% 20. Diabetes mellitus; 30% vs. 30% vs. 40% vs. 56% vs. 100% 21. Dyslipidaemia; data not shown 22. Smoker; 10% vs. 20% vs. 0% vs. 0% vs. 10% 23. Coronary artery disease; data not reported 24. Atrial fibrillation; data not reported		EV in stroke or TIA: IS and TIA
	Clinical severity; 1. NIHSS 17.5 2. NIHSS 2 3. NIHSS 0 4. NIHSS 5.5 5. NIHSS 0		Main finding: The study demonstrated the potential of exploring EV protein cargo from low plasma volumes using proteomics and that the protein content reflected specific subtypes of stroke. More research to unearth their potential as biomarkers is needed.
Gaceb et al. (2024)	Patient group Ischemic stroke Mean age = 79 (56–91) 59% F, 41% M *N* = 39 Control group Mean age = 66 (26–96) 38% F, 62% M *N* = 39	To evaluate the following 4 aims: 1. provide a quantitative timeline of blood‐based pericyte derived EV secretion after stroke. 2. Characterise the protein cargo of pericyte‐derived EV. 3. Identify the vascular signalling pathways implicated by the protein content of pericyte‐derived EV. 4. Examine whether any other clinical parameters impact on the release of pericyte‐derived EV after stroke.	EV concentration: pericyte‐derived EVs (CD140b+) significantly increased at 12–24 h (*p* = 0.0041), 2–6 days (*p* = 0.02) compared with controls. No difference at 0–6 h after IS symptom onset.
			Sampling time point: 1. <1 day 2. 2–6 days
	Treatment status; data not shown		EV size: Data not reported.
	Risk factors (stroke vs. control) 1. Hypertension; 62% vs. 77% 2. Diabetes mellitus; 33% vs. 10% 3. Dyslipidaemia; data not shown 4. Smoker; 3% vs. 3% 5. Coronary artery disease; data not reported 6. Atrial fibrillation; 10% vs. 0%		EV content: Protein in CD140b+ EV (pericytes) was measured. Hyperacute (0–6 h); Protein was decreased in pericyte EV compared with controls; SIRT2 (*p* = 0.002), CD40 (*p* = 0.00005), FGF21 (fibroblast growth factor) (*p* = 0.002), GDNF (glial cell neurotrophic factor) (*p* = 0.004), CD244 (*p* = 0.008), MCP‐4 (*p* = 0.01), IL17 (*p* = 0.014), IL5 (*p* = 0.028), IL6 (*p* = 0.037), IL2 (*p* = 0.049). Mainly decreased production of inflammatory related molecules. 12–24 h; Increased CD244 (*p* < 0.0001), MCP‐4 (*p* < 0.0001), MMP1 (*p* < 0.0001), CXCL5 (*p* < 0.0001), CD40 (*p* < 0.0001), VEGF (*p* = 0.0002), PDL1 (programmed death ligand 1) (*p* = 0.0002), CXCL6 (*p* = 0.0007), CD5 (*p* = 0.01), upa (urinkinase type plasmingogen activator (*p* = 0.04), MCP‐2 (*p* = 0.047). 2–6 days; MMP‐1 (*p* = 0.011), CD40 (*p* = 0.0178), VEGF‐a (*p* = 0.025), CXCL5 (*p* = 0.027). For 12–24 and >2 days; mainly increase in production of proteins related to angiogenesis, vascular remodelling and inflammation.
	Clinical severity; NIHSS = 7		EV in stroke or TIA: IS
			Main finding: Significantly increased pericyte EV at 12–24 h and 2–6 days post‐stroke compared with controls. Early and time‐dependant increase in pericyte‐derived EV, proteins switch to being pro‐angiogenic and pro‐inflammatory over time.
Simak et al. (2006)^a^	Patient group total All stroke Mean age = 79 (62–83) 36%F, 64% M *N* = 41 Patient Subgroup 1 Moderate to severe stroke patients Mean age = 81 (64–84) 29% F, 71% M *n* = 21 Patient Subgroup 2 Mild stroke patients Mean age = 76 (57–82) 40% F, 60% M *n* = 20 Age matched healthy control group Mean age: 70 (57–81) 25% F, 75% M *N* = 23	To assess whether endothelial EVs are increased in patients with IS and are correlated with stroke severity, brain lesion volume and outcome.	EV concentration: PS + EV concentration was significantly higher in IS patients (*p* = 0.002).
			Sampling time point: 1. Between 1 and 2 days
	Treatment status: Data not reported		EV size: Data not reported.
			EV content: All four endothelial EV phenotypes were elevated in subgroup of moderate‐severe stroke patients relative to controls (all *p* < 0.05) endoglin‐positive endothelial EV: CD105+ CD41a+ CD45+) (E+), specific endothelial EV expressing VE‐cadherin and endoglin: CD105+CD144+ (C+), EV expressing phosphatidylserine: CD105+PS+ CD41a) (PS+) and EV expressing ICAM‐1: CD105+CD54+ CD45) (I+). No difference was seen in platelet (CD41a+), red blood cell EV (CD235a+) or white blood cell EV (CD45+) between acute stroke and control groups. In the patients with acute ischemic stroke three endothelial EV phenotypes (E+, PS+ and I+) correlated significantly with brain lesion volume, with I+ (*p* = −0.002) showing the strongest correlation.
	Risk factors (all stroke vs. control) 1. Hypertension; 62% vs. 38% 2. Diabetes mellitus; 24% vs. 13% 3. Dyslipidaemia 51% vs. 63% 4. Smoker; 48% vs. 38% 5. Coronary artery disease; 27% vs. 25% 6. Atrial fibrillation; 32% vs. 0%		EV in stroke and TIA: IS
	Clinical severity; 1. NIHSS <5 2. NIHSS ≥5		Main finding: Certain circulating endothelial EV phenotypes may be associated with severity, lesion volume and outcome of acute ischemic stroke.
Chen et al. (2015)	Patient Group 1 Large artery atherosclerosis (LAA) Mean age = 68 40% F, 60% M *N* = 53 Patient Group 2 Small artery occlusion (SAO) Mean age = 68 44%F, 56% M *N* = 59 Healthy control group Mean age = 66 42.86%F, 57.14% M *N* = 35	To assess levels of circulating platelet EV and other platelet parameters (mean platelet volume, platelet count, plateletcrit and platelet distribution width in acute ischaemic stroke patients.	EV concentration: Data not reported.
			Sampling time point: 1. <2 days
	Treatment status: Aspirin and cilostazol		EV size: Data not reported.
			EV content: Levels of circulating platelet EV concentration were significantly elevated in AIS patients (LAA and SAO) when compared to healthy controls, but LAA and SAO did not significantly differ to each other.
	Risk factors (LAA vs. SAO vs. control) 1. Hypertension; 43% vs. 53% vs. 12% 2. Diabetes mellitus; 13% vs. 14% vs. 1% 3. Dyslipidaemia; data not shown 4. Smoker; 14% vs. 8% vs. 7% 5. Coronary artery disease; data not reported 6. Atrial fibrillation; data not reported		EV in stroke or TIA: IS
	Clinical severity: data not shown		Main finding: Circulating platelet EV was significantly elevated in IS patients and correlated with infarct volume in the LAA subtype.
Jung et al. (2009)^a^	Patient group IS Mean age = 66 38.4% F, 61.6% M *N* = 73 Risk factor matched control group Mean age = 65 34.9%F, 65.1% M *N* = 275	Investigated circulating endothelial EV profiles in patients with acute IS. Also, the study examined the degree to which Endothelial EV levels are associated with cerebral artery stenosis and the importance of EV profiling for the diagnosis of intracranial arterial stenosis (ICAS) and extracranial arterial stenosis (ECAS).	EV concentration: Total EV showed no difference between IS and risk factor group.
			Sampling time point: 1. 2.8 days median (<7 days)
	Treatment status; data not shown		EV size: Data not reported.
			EV content: Levels of CD62+ endothelial EV were strongly associated with stroke severity (*p* = 0.006) and infarct volume (*p* = 0.001).
	Risk factors (stroke vs. control) 7. Hypertension; 71% vs. 79% 8. Diabetes mellitus; 36% vs. 28% 9. Dyslipidaemia; 26% vs. 23% 10. Smoker; 18% vs. 19% 11. Coronary artery disease; 15% vs. 11% 12. Atrial fibrillation; 15% vs. 5%		EV in stroke or TIA: IS and vascular risk factors (no events) control group.
	Clinical severity; 1. NIHSSS 0–5 = 77% 2. NIHSS 6–13 = 18% 3. NIHSS ≥14 = 5%		Main finding: Circulating endothelial EVs are markers of vascular pathology and risk of stroke.
Switonska et al. (2015)	Patient group IS Mean age = Mean not reported (36–93) 42% F, 58% M *N* = 73 Control group Mean age = Mean not reported (33–67) Gender not reported. *N* = 30	To evaluate the procoagulant activity of EV and levels of tissue‐factor‐bearing EV (EV‐TF), tissue factor (TF) and tissue factor pathway inhibitor (TFPI) in patients with acute IS.	EV concentration: The median concentration of EV in IS was higher than that of controls (*p* = 0.06).
			Sampling time point: 1. 1 day 2. 7 days
	Treatment status; data not shown		EV size: Data not reported.
			EV content: EV‐TF were significantly higher in stroke subjects (*p*<0.001).
	Risk factors (stroke only) 1. Hypertension; 75% 2. Diabetes mellitus; 26% 3. Dyslipidaemia; data not shown 4. Smoker; 32% 5. Coronary artery disease; data not shown 6. Atrial fibrillation; data not shown		EV in stroke or TIA: IS
	Clinical severity; NIHSS = 8.38		Main finding: IS patients have increased generation of TF‐bound EV.
Burrello et al. (2021)	Patient group TIA (investigated TIA symptoms) Mean age = 69 52.5% F, 47.5% M *N* = 40 Healthy control group Mean age = 64 50% F, 50% M *N* = 20	To investigate the profile of EV‐surface antigens in patients with symptoms suspicious for TIA.	EV concentration increased in TIA group (*p* < 0.001) compared to controls by nanosight tracking analysis and by expression of tetraspanins (CD9, CD81 and CD63).
			Sampling time point: 1. <2 days
	Treatment status; data not shown		EV size: Data not reported.
			EV content: Multiplex flow cytometry showed higher mean fluorescent intensities in TIA patients for CD62P, CD42A, MCSP, CD44, CD326, CD142, CD14, CD8 and CD2 when compared to control (*p* < 0.05).
	Risk factors (control vs. TIA) 1. Hypertension; 45% vs. 65% 2. Diabetes mellitus; 5% vs. 5% 3. Dyslipidaemia; 65% vs. 85% 4. Smoker; 25% vs. 23% 5. Coronary artery disease; data not shown 6. Atrial fibrillation; data not shown		EV in stroke or TIA: TIA
	Clinical severity; data not shown		Main finding: EV surface antigens are different in patients with TIA.
Lundstrom et al. (2020)	Patient group IS and/or TIA Mean age = 72 42%, 58% M *N* = 211 total *N* = 72 TIA *N* = 139 IS Healthy control group Mean age = 70 42% F, 58% M *N* = 53	Characterise the subtypes of platelet EV (TF+) in IS and TIA. To determine whether there is an association with patient outcome.	EV concentration: Data not reported.
			Sampling time point: 1. 14 days 2. 30 days
	Treatment status; data not shown		EV size: Data not reported.
			EV content: Activated platelet EV populations (P‐selectin and PS_+_) and procoagulant (tissue factor+) were higher in IS and TIA vs. controls.
	Risk factors (stroke only) 1. Hypertension; 64% 2. Diabetes mellitus; 15% 3. Dyslipidaemia; data not shown 4. Smoker; 18% 5. Coronary artery disease; data not shown 6. Atrial fibrillation; data not shown		EV in stroke or TIA: IS and TIA
	Clinical severity; data not shown		Main finding: Identified higher levels of circulating procoagulant EV in TIA and IS patients.
Kowalski et al. (2023)	Patient group Confirmed stroke Mean age = 67 37.5% F, 62.5% M *N* = 8 Control group Stroke mimic Mean age = 74 85.7% F, 14.3% M *N* = 7	This is a proof‐of‐concept study, ultra‐early blood (in ambulance/mobile stroke unit) and EV markers of neuroinflammation, cerebral insult and outcome in acute stroke. The bloods were compared from at the time of entry to mobile stroke unit and 24 h later, the outcomes measured were EV biomarker concentrations.	EV concentration: Data were not reported.
			Sampling time point: 1. 0 day 2. 1 day
	Treatment status; 1. Thrombolysis; 75% stroke group 2. Thrombectomy 25% stroke group		EV size: Data were not reported.
			EV content: matrix–matrix metalloproteinase‐9 (MMP‐9), platelet factor 4 (CXCL‐4), C‐reactive protein (CRP), interleukin‐6 (IL‐6), osteopontin (OPN) and platelet/endothelial cell adhesion molecule‐1 (PECAM‐1) were elevated in EVs (*p* < 0.01).
	Risk factors; data not shown		EV in stroke or TIA details: IS (8/30 diagnosed using imaging).
	Clinical severity; 1. NIHSS stroke; 11 2. NIHSS Mimic; 6		Main finding: Inflammatory cascade is activated and mimicked in the EVs in as early as 36 min after stroke.
Pawelczyk et al. (2017)	Patient Group1 normal lipid, normal glycaemia. Mean age = 76 56% F, 44% M *N* = 25 Patient Group 2 IS with normal lipid, hyperglycaemia Mean age = 73 48% F, 52% M *N* = 21 Patient Group 3 IS with hyperlipidaemia, normal glycaemia Mean age = 66 67% F, 33% M *N* = 27 Patient Group 4 IS with hyperlipidaemia, hyperglycaemia Mean age = 70 38% F, 62% M *N* = 21 Control group Mean age = 60 52.4% F, 47.6% M *N* = 21	To explore the relationship between hyperlipidaemia and hyperglycaemia and platelet EV in IS patients.	EV concentration: Data not reported.
			Sampling time point: 1. 1 day
	Treatment status; acetylsalicylic acid		EV size: Data not reported.
	Risk factors; data not shown		EV content: Platelet EVs (CD61+) are significantly higher in acute IS (*p* < 0.001) compared to healthy controls. Percentages of P‐EV were higher in all groups of stroke patients compared to HCs.
	Clinical severity; data not shown		EV in stroke or TIA: IS
			Main finding: Elevated levels of platelet EV were observed in stroke patients, which adds evidence to determine the important role of platelet EV in coagulation and their thrombogenic potential in terms of IS.
He et al. (2017)	Patient group IS Mean age = 62 37% F, 63% M *N* = 76 Control group Mean age = 63 *N* = 70	To determine leukocyte EV (LEV) in IS compared to control and the utility of LEV as a proinflammatory mediator of vascular inflammation and as a biomarker of severity and outcome of IS.	EV concentration: Data not reported.
			Sampling time point: 1. <7 days
	Treatment status; data not reported		EV size: Data not reported.
	Risk factors (stroke only vs. control) 1. Hypertension; 60% vs. 58% 2. Diabetes mellitus; 17% vs. 25% 3. Dyslipidaemia; 47% vs. 39% 4. Smoker; 37% vs. 40% 5. Coronary artery disease; data not shown 6. Atrial fibrillation; data not shown		EV content: CD45+ LEV (*p* = 0.027), CD14+ monocyte‐derived EV (*p* = 0.013), CD4+ lymphocyte‐derived EV (*p* = 0.038), CD15+ granulocyte‐derived EV (*p* = 0.021) levels in patient group were significantly increased than those in the control group. Levels of CD14+ monocyte‐derived EV were significantly correlated with stroke severity (*r* = 0.355, *p* = 0.019), cerebral vascular stenosis severity (*r* = 0.255, *p* = 0.025) and stroke subtypes (*r* = 0.242, *p* = 0.036).
	Clinical severity; 1. NIHSS < 5, *n* = 35 2. NIHSS ≥ 5, *n* = 41		EV in stroke or TIA: IS
			Main finding: Levels of CD14+ LMPs were correlated with stroke severity, cerebral vascular stenosis severity.
Li et al. (2015)	Patient group IS Mean age = 64 52% F, 48% M *N* = 68 Control group Mean age = 60 34.43% F, 65.57% M *N* = 61	Quantify endothelial and platelet EV in IS compared to control. Determine if endothelial EV can be used as a marker of endothelial dysfunction and as a biomarker of stroke severity.	EV concentration: Data not reported.
			Sampling time point: 1. <7 days
	Treatment status; data not shown		EV size: Data not reported.
	Risk factors (stroke only vs. control) 1. Hypertension; 51% vs. 66% 2. Diabetes mellitus; 18% vs. 25% 3. Dyslipidaemia; 84% vs. 85% 4. Smoker; 34% vs. 32% 5. Coronary artery disease; data not shown 6. Atrial fibrillation; data not shown		EV content: Levels of circulating endothelial CD144+/CD41a−, CD31+ CD41a−, CD62E+, and Annexin V+, CD62E+ EV, but not platelet EV, were significantly increased in acute ischemic stroke patients, compared with control subjects (*p* < 0.05).
	Clinical severity;1. NIHSS < 5, *n* = 38 NIHSS ≥ 5, *n* = 30		EV in stroke or TIA: IS
			Main finding: Identified endothelial EV that may be effectively utilised as biomarkers of stroke severity in acute ischemic stroke patients.
Lu et al. (2023)	Patient group IS Mean age = 68. 57% F, 43% M *N* = 75 Control group Mean age = 68 42.67% F, 57.33% M *N* = 75	To determine Silent Information Regulator 2 (SIRT2) protein expression in serum EV for IS vs. non‐IS (role in diagnosis and prognosis).	EV concentration: Data not reported.
			Sampling time point: 1. 1 day 2. 90 days
	Treatment status; data not shown		EV size: Data not reported.
	Risk factors (stroke only vs. control) 1. Hypertension; 41% vs. 65% 2. Diabetes mellitus; 17% vs. 19% 3. Dyslipidaemia; data not shown 4. Smoker; 17% vs. 32% 5. Coronary artery disease; data not shown 6. Atrial fibrillation; data not shown		EV content: SIRT2 protein concentration in serum EV was higher in AIS patients than controls (*p* < 0.001). Additionally, higher SIRT2 concentration of serum EV was associated with National Institutes of Health Stroke Scale (NIHSS) *d* ≥ 4 (*p* < 0.001) and modified Rankin scale (mRS) ≥3 (*p* = 0.025) in AIS patients.
	Clinical severity; NIHSS ≥ 4		EV in stroke or TIA: IS
			Main finding: SIRT2 is elevated in serum EV and may be a valuable biomarker of IS.
Kim et al. (2012)^a^	Patient group IS Mean age = 66 41% F, 59% M *N* = 111 Control group Mean age = 60 62% F, 38% M *N* = 50	To determine circulating CD105+ and CXCR4+ EV levels and apoptotic microparticle levels in stroke.	EV concentration: Data not reported.
			Sampling time point: 1. Mean 3.21 days
	Treatment status; data not shown		EV size: Data not reported.
	Risk factors (stroke only vs. control) 1. Hypertension; 60% vs. 48% 2. Diabetes mellitus; 31% vs. 22% 3. Dyslipidaemia; 13% vs. 12% 4. Smoker; data not shown 5. Coronary artery disease; data not shown 6. Atrial fibrillation; 12% vs. 4%		EV content: In IS patients, the levels of CD105+/AV− microparticles were increased as the baseline stroke severity increased (*p* = 0.028 for initial DWI lesion volume, *p* = 0.005 for initial NIHSS score). The proportion of CD105+/CXCR4+ EV among CXCR4+ EV was higher in IS patients than in healthy subjects (*p* = 0.011).
	Clinical severity; data not shown		EV in stroke or TIA: IS
			Main finding: A significant relationship between circulating CD105+/AV− microparticles and the extent of infarct was identified.
Picciolini et al. (2023)	Patient group IS Median age = 74 42% F, 58% M *N* = 19 Control group Median age = 66 50% F, 50% M *N* = 20	To investigate brain and non‐brain associated proteins in circulating EV from stroke patients in subacute phase.	EV concentration: no difference in mean EV concentration between HC and IS (*p* > 0.05).
			Sampling time point: 1. Median 21 days
	Treatment status; data not shown		EV size: Mean EV size did not change in controls vs. IS.
	Risk factors; data not shown		EV content: There was a significant decrease in CD106+ EVs in IS compared to control (*p* < 0.01).
	Clinical severity; Median modified Barthel Index = 30		EV in stroke or TIA: IS
			Main finding: Detected multiple subpopulations of EVs by specific tissue markers expressed on the vesicle surface. This study had a small sample size; however, the results warrant a larger study to investigate EVs as biomarkers of tissue damage and recovery after IS.
Edwardson et al. (2024)	Patient group IS Median age = 66 45% F, 55% M *N* = 58 Risk factor control group Median age = 66 48% F, 52% M *N* = 46	To identify alterations in the quantity of plasma brain‐derived EVs (astrocyte [ADE], neurons [NDE] and oligodendrocytes [ODE] derived EVs) over the first month post‐stroke.	EV concentration: Data not reported
			Sampling time point: 1. 5 days 2. 15 days 3. 30 days
	Treatment status; 28% thrombolysis, 12% thrombectomy		EV size: Data not reported.
	Risk factors (stroke vs. control) 1. Hypertension; 83% vs. 80% 2. Diabetes mellitus; 40% vs. 37% 3. Dyslipidaemia; 45% vs. 85% 4. Smoker; 24% vs. 22% 5. Coronary artery disease; data not reported 6. Atrial fibrillation; 10% vs. 7%		EV content: Levels of ADEs (CD9+ EAAT1+) were significantly higher in stroke patients compared to controls at 5‐ (*p* < 0.002), 15‐ (*p* < 0.002) and 30‐ (*p* < 0.005) days post‐stroke. No significant difference for NDE (CD9+ L1CAM+, CD171+) and ODEs (CD9+ MOG+) compared to controls at all timepoints.
	Clinical severity; NIHSS = 4.3		EV in stroke or TIA: IS
			Main finding: ADE levels are elevated 5–30 days post‐IS and may provide potential as biomarkers of BBB breakdown due to associations between ADE levels and lesion size.
Escudero‐Guevara et al. (2025)	Patient Group 1 Mild clinical severity Median age = 71 22% F, 78% M *N* = 9 Patient Group 2 Severe clinical severity Median age = 68 33% F, 67% M *N* = 9 Control group Median age = 72 22% F, 78% M *N* = 9	To characterise the inflammatory and angiogenic components of circulating EVs in AIS patients.	EV concentration: Mild clinical severity strokes had significantly fewer plasma EVs than controls (*p* = 0.011). Plasma EV concentration in the severe group was not statistically different to controls.
			Sampling time point: 1. Between 1 and 2 days
	Treatment status; data not shown		EV size: Plasma EVs in the severe group were significantly larger than those in the mild clinical severity stroke group (*p* = 0.0052). Plasma EVs in the mild clinical severity group were smaller than control EVs (*p* = 0.046).
	Risk factors (Group 1 vs. Group 2 vs. control) 1. Hypertension; 56% vs. 100% vs. 44% 2. Diabetes mellitus; 56% vs. 66% vs. 11% 3. Dyslipidaemia; data not shown 4. Smoker; data not shown 5. Coronary artery disease; data not shown 6. Atrial fibrillation; data not shown		EV content: Plasma EVs from both mild and severe stroke patients exhibited significantly higher TNF‐ (*p* < 0.05) and nitrotyrosine (*p* < 0.05) but lower levels of PIGF (*p* < 0.05) than EVs from the control group. EVs from severe‐stroke patients had higher VEGF levels compared to mild clinical severity (*p* < 0.05) and control groups (*p* < 0.01). Mild severity stroke had higher levels of IL‐6 in EVs than control (*p* < 0.01) but not severe.
	Clinical severity; 1. NIHSS = 3.8 Group 1 2. NIHSS = 9.2 Group 2		EV in stroke or TIA: IS
			Main finding: The study identified distinct proinflammatory and angiogenic profiles in EVs from mild‐ and severe‐stroke patients. Specifically, EVs from mild stroke patients had higher levels of IL‐6 and severe cases exhibited elevated VEGF levels. The exact role of IL‐6 in stroke pathology remains unclear.
Williams et al. (2007)^a^	Patient group IS Mean age = 66 50% F, 50% M *N* = 10 Control group Stroke Mimics Mean age = 66. 50% F, 50% M *N* = 10	Comparison of endothelial EV in IS and stroke mimic and to determine if increases in endothelial EV are related to activation or apoptosis.	EV concentration: Data not reported.
			Sampling time point: 1. 1 day
	Treatment status; data not shown		EV size: Data not reported.
	Risk factors (stroke vs. Mimic) 1. Hypertension; 80% vs. 80% 2. Diabetes mellitus; 30% vs. 30% 3. Dyslipidaemia; 50% vs. 50% 4. Smoker; data not shown 5. Coronary artery disease; 20% vs. 20% 6. Atrial fibrillation; 10% vs. 0%		EV content: Endothelial EV levels are similar in patients with IS and stroke mimics. Expression of PECAM‐1, *p* = 0.393; E‐selectin, *p* = 0.579. PECAM‐1/E‐selectin ratio was >4 = generated by activation and not apoptosis/necrosis. No difference between groups.
	Clinical severity; data not shown		EV in stroke or TIA: IS and stroke mimics.
			Main finding: The results suggest that endothelial EV may not be a specific marker for IS, given the inability to discriminate between stroke mimics and IS.
Agouni et al. (2019)	Patient Group 1 IS Mean age = 51 4.5% F, 95.5% M *N* = 66 Patient Group 2 TIA Mean age = 49 4% F, 96% M *N* = 21 Control group Mean age = 48 25% F, 75% M *N* = 24	Aimed to count medium size EV and phenotype them according to cellular origin in TIA and IS. Determine EV concentrations and character in control, TIA and IS groups.	EV concentration: no difference in total EVs between the 3 groups (events/ul) p>0.05) at onset of attacks, or at 5 days post‐event. However, circulating EV levels increase post‐incident at both 5 and 30 days vs. onset concentration (*p*<0.05) in both TIAs and AIS at 30 days post‐incident.
			Sampling time point: 1. 2 days 2. 5 days 3. 30 days
	Treatment status; 1. TIA 10% thrombolysis 2. Stroke 18% thrombolysis		EV size: Data not reported.
	Risk factors (control vs. TIA vs. stroke) 1. Hypertension; 0% vs. 62% vs. 66% 2. Diabetes mellitus; data not shown 3. Dyslipidaemia; 0% vs. 71% vs. 58% 4. Smoker; 0% vs. 29% vs. 44% 5. Coronary artery disease; 0% vs. 10% vs. 9% 6. Atrial fibrillation; 0% vs. 5% vs. 5%		EV content: Control, TIA and AIS groups. Increase in endothelial EVs in TIA vs. HC (*p* = 0.0156) and in IS vs. TIA (*p* = 0.0007). TIA and IS patients not only show increased endothelial EV (CD146+), but also activated endothelial EV (CD62e+), activated platelet EV (CD62p+), granulocytes EV (CD66b+), leukocytes EV (CD45+) and erythrocytes EV (CD235A+) (*p* < 0.05 for all in TIA, *p* < 0.001 for all in IS). Protein surface content at onset, 5 and 20 days. Increased activated endothelial EV in IS patients at both 5‐ and 30‐days vs. onset. *p* < 0.05. Both platelet and endothelial EV were increased in TIA and AIS at 5 and 30 days (*p* < 0.05). Activated platelets increased in both TIA and IS at 5 and 30‐days (*p* < 0.05). On the contrary, erythrocyte EV significantly decreased in both TIA and IS at 5 and 30 days (*p* < 0.01 for all). Granulocyte EV were significantly higher at 5 and 30‐days in TIA vs. onset (*p* < 0.05) and higher at 30 days in IS (*p* < 0.05). Annexin V+ EV significantly increased at 30‐days post‐TIA vs. onset (*p* < 0.01) and were significantly higher at 5‐ and 30‐days post‐AIS vs. onset (*p* < 0.05).
	Clinical severity; 1. NIHSS TIA = 1.67 2. NIHSS stroke = 3.35		EV in stroke or TIA: TIA and IS
			Main finding: Increased EVs from activated endothelial cells, platelets, granulocytes, leukocytes and pro‐coagulant EVs (AV+) at 5‐ and 30‐days post‐ischaemic event and may contribute to the elevated risk of stroke in this population.
Forro et al. (2024)	Patient group IS Median age = 66 44% F, 56% M *N* = 18 Control group Median age = 65 44% F, 56% M *N* = 9	To characterise the ADEV profile of IS patients, focussing on GFAP as a cargo protein in EVs.	EV concentration: Data not reported.
			Sampling time point: 1. 1 day 2. 7 days 3. 30 days
	Treatment status; 50% Thrombolysis		EV size: Data not reported.
	Risk factors; data not shown		EV content: Higher expression of GFAP in EV from stroke patients isolated at Days 1 and 7 of event compared to controls (*p* < 0.05 for both).
	Clinical severity; NIHSS = 7.5		EV in stroke or TIA: IS
			Main finding: Post‐stroke ADEV GFAP levels were elevated at D1 and D7 but not M1 compared to controls and may dynamically reflect changes during the first month post‐ischaemia.
Forro et al. (2024)	Patient group IS Median age = 66 44% F, 56% M *N* = 18 Control group Median age = 65 44% F, 56% M *N* = 9	To assess levels of AQP4 and GDNF in EVs from IS patients at 3 intervals post‐event.	EV concentration: Data not reported
			Sampling time point: 1. 1 day 2. 7 days 3. 30 days
	Treatment status; 50% Thrombolysis		EV size: Data not reported.
	Risk factors (stroke vs. control) 1. Hypertension; 100% vs. 66% 2. Diabetes mellitus; 50% vs. 44% 3. Dyslipidaemia; 22% vs. 22% 4. Smoker; 17% vs. 22% 5. Coronary artery disease; data not reported 6. Atrial fibrillation; 17% vs. 11%		EV content: Levels of AQP4 and GDNF were significantly higher in stroke patients at 24 h post‐event compared to controls (*p* < 0.05).
	Clinical severity; NIHSS = 7.5		EV in stroke or TIA: IS
			Main finding: Post‐IS, EV levels of AQP4 and GNDF were higher at D1 compared to controls and may hold promise for the development of biomarkers in IS.
Slomka et al. (2017)	Patient group IS* Median age = 69 44%F, 56% M *N* = 66 *Group split between high (*n* = 38) and low (*n* = 28) CRP levels.	Investigate if haemostatic factors (EV, EV, TF and TFPI) account for worse outcome in IS and if these are associated with elevated levels of CRP.	EV concentration: Data not reported.
			Sampling time point: 1. 0 day 2. 7 days
	Treatment status; 27% Alteplase		EV size: Data not reported.
	Risk factors (stroke only) 1. Hypertension; 76% 2. Diabetes mellitus; 27% 3. Dyslipidaemia; data not shown 4. Smoker; 35% 5. Coronary artery disease; data not reported 6. Atrial fibrillation; data not reported		EV content: Tissue factor‐positive EV was not associated with levels of CRP, *p* > 0.05.
	Clinical severity; 1. CRP≥3 NIHSS = 9 2. CRP<3 NIHSS = 5.5		EV in stroke or TIA: IS
			Main finding: There is no inflammation effect of haemostatic factors present in this study as shown by no correlation between CRP and platelet EV.
Schrick et al. (2022)	Patient group IS Mean age = 66 34% F, 66% M *N* = 18 Control group Mean age = 57 50% F, 50% M *N* = 20	Prospective study assessing differences in EV origin following IS.	EV concentration: total EV is increased post‐stroke vs. HC (*p* < 0.001).
			Sampling time point: Data not reported.
	Treatment status; All IS = clopidogrel		EV size: Data not reported.
	Risk factors; data not shown		EV content: CD31+ EV increased post‐stroke vs. HC (*p* < 0.05)—endothelial (CD42A+) EVs increased post‐stroke vs. HC (*p* < 0.0001).
	Clinical severity; Data not shown		EV in stroke or TIA: IS
			Main finding: Platelet function correlates with EV in patients on anti‐platelet medication. Platelet EV is associated with residual platelet reactivity.

^a^ Studies published before 2014 (pre‐MISEV) could not receive an additional point allocated for explicit citation of EV reporting guidelines.

### 3.2 Study Ranking

**FIGURE 3:**
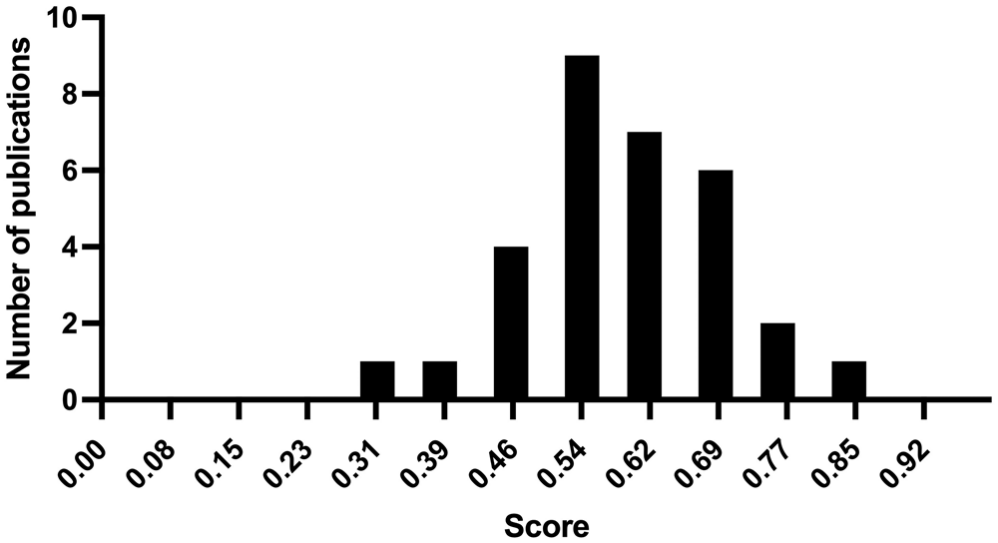
The number of articles for each scoring rank.

The methodological appraisal score was assessed across all studies as described in section 2.3. The article scores were deemed within a range of 0.31 to a maximum of 0.84 for the highest scoring study (Figure 3). The highest scoring study assessed (Otero‐Ortega et al. 2021), marked 0.84, reached all characterisation parameters and isolated EVs using a preferred technique of SEC. However, the study did not mention the ISEV guidelines and thus lost a mark. Additionally, EV characterisation for this study was carried out via several techniques, including, nanoparticle tracking analysis, western blotting and enzyme linked immunosorbent assays (ELISAs). The population sizes for each group were large; however, the study only had one control group. Two studies scored 0.76 and both had large population sizes and used a gold standard diagnosis technique; however, EV isolation and characterisation techniques were not robust (Yao et al. 2017; Datta et al. 2014). Six studies scored 0.69 (Cappelletti et al. 2022; Rosińska et al. 2019; Chiva‐Blanch et al. 2016; Eyileten et al. 2022; Maciejewska‐Renkowska et al. 2024; Reymond et al. 2025) and scored highly (3/3) for population sizes but also lost marks on EV isolation and characterisation techniques. Of all 31 assessed studies, the majority scored either 0.54 (Escudero‐Guevara et al. 2025; Kowalski et al. 2023; Pawelczyk et al. 2017; He et al. 2017; Li and Qin 2015; Lu et al. 2023; Kim et al. 2012; Picciolini et al. 2023; Edwardson et al. 2024) or 0.61 (Gaceb et al. 2024; Simak et al. 2006; Chen et al. 2015; Jung et al. 2009; Świtońska et al. 2015; Burrello et al. 2021; Lundström et al. 2020). All studies lost marks for the EV isolation and characterisation methods. Four of the studies assessed were given lower scores, four at 0.46 (Williams et al. 2007; Agouni et al. 2019; Forró et al. 2024; Forró et al. 2024), one 0.38 (Słomka et al. 2017) and one at 0.31 (Schrick et al. 2022). The studies overall were shown to be less robust in recruitment, diagnosis and EV isolation and characterisation. A more detailed breakdown can be seen in Table S2, the papers are organised from highest to lowest score in the main table (Table 1).

### 3.3 EV Concentration

Total EV concentration was reported in 9 of 31 studies and included both TIA and IS groups. According to six of the studies, with a total of 277 IS patients, there was no significant difference in total EV concentration between IS and control groups (*p* > 0.05) (Escudero‐Guevara et al. 2025; Otero‐Ortega et al. 2021; Reymond et al. 2025; Picciolini et al. 2023; Jung et al. 2009; Agouni et al. 2019). However, in two studies for a total of 91 IS patients, the EV concentration was found to be higher in IS patients in comparison to healthy controls (HCs) (*p* < 0.06 and *p* < 0.001) (Świtońska et al. 2015; Schrick et al. 2022). A meta‐analysis could not be performed due to the differences in techniques used for EV isolations, and based on this evidence EV number does not seem a viable biomarker for IS.

EV concentration post‐TIA was reported by three studies but gave contradicting results. The first study showed total EV concentration was significantly higher in suspected TIA patients at onset compared to HC (*p* < 0.001) (Burrello et al. 2021). On the contrary, the second study reported total plasma‐derived EV at the onset of TIA was no different to HC at onset (Agouni et al. 2019). However, the same study reported EV concentration at 30 days post‐TIA was significantly higher when compared to HC (*p* < 0.05). Moreover, another study reported no difference between EV concentrations in TIA patients compared to control subjects (Reymond et al. 2025); however, this study was carried out on a small population of patients (*N* = 10) and should be taken into consideration.

### 3.4 EV Size

Changes in EV size can often indicate disease stage (Badovinac et al. 2021), which is highly important in diagnosis. Many of the studies included in this systematic review focused on EV biological cargo only and consequently did not compare the EV size between TIA/IS stroke and control groups. Surprisingly, only two studies reported on EV size in IS (Escudero‐Guevara et al. 2025; Picciolini et al. 2023). According to the first study, EV dimensions were of similar size when isolated from the serum of stroke patients and control group. All EVs analysed in this study would be classified as smaller EVs with a mean size of 147.9 ± 25.7 nm for stroke patients and 150 ± 24.5 nm for HC. It is of note that this study scored highly on the EV isolation methodology category, with EVs isolated using the preferred SEC technique and are therefore compliant with the latest recommendations, enabling robust comparison with future studies. The second study compared mild and severe clinical severity IS patients with controls and reported significantly larger EVs were observed in the severe group compared to the mild group (*p* < 0.01). However, the mild clinical severity group were reported to have significantly smaller EVs than the control group (*p* < 0.05).

According to the only two studies that reported on mean EV size in patients with suspected TIA, significantly smaller EVs were isolated from the plasma of TIA patients compared to HC in one study(*p* < 0.01) (Burrello et al. 2021). However, the second study reported no significant difference in the size of EVs isolated from TIA patients and controls (*p* > 0.05); it should be noted that this study was comprised of small sample sizes in each group (*n* = 10) and suggest larger numbers per group is needed for robust comparison (Escudero‐Guevara et al. 2025). Importantly, in more recent years, flow cytometry has been used to characterise EVs, which is fundamentally based on particle size; therefore it is important that EV size is accurately assessed to assure appropriate and robust techniques are applied.

### 3.5 EV Cell Origin

The main aim of this review was to uncover EV origin and function in TIA and IS. The majority of the studies focussed on surface molecule expression as a means of identifying this. The main components that were studied included characteristics of platelets, endothelial cells (ECs), neural cells, erythrocytes and white blood cells, such as leukocytes, lymphocytes and granulocytes. Each cell type will be discussed individually.

#### 3.5.1 Platelets

One study focussing on circulating platelet microparticles in IS reported no difference in total number of platelet (CD61+) EV (*p* > 0.05) between stroke subjects, HC, large artery atherosclerosis (LAA) and small artery occlusion (SAO) stroke subjects (Rosińska et al. 2019). However, another study reported higher levels of circulating platelet microparticles in LAA (*p* < 0.05) and SAO (*p* < 0.05) compared to HC. There was however no difference between LAA and SAO reported (*p* > 0.05) (Chen et al. 2015). Discrepancies in results could be due the type of stroke and timing of sample collection and analysis post‐incident as another study reported no difference in platelet EV (CD41+) concentration between AIS and HC at stroke onset (*p* > 0.05). However, at 5‐ and 30‐days post‐stroke platelet EV were reported to be significantly higher (Agouni et al. 2019). This is supported by another five studies which also reported a significant increase in platelet EV [CD61+ (Chiva‐Blanch et al. 2016; Eyileten et al. 2022; Pawelczyk et al. 2017; Maciejewska‐Renkowska et al. 2024)], [CD42A+ (Schrick et al. 2022)] in IS patients compared with HC (*p* < 0.05 for all).

A study reporting on TIA showed no difference in platelet EV concentration (CD61+) between patients and HCs at onset (Agouni et al. 2019). Moreover, at 5‐ and 30‐days post‐TIA the study reported that significantly higher platelet EV. Another study reported that patients with suspected TIA had higher platelet EV (CD142+) than HC (Burrello et al. 2021).

#### 3.5.2 Endothelial Cells

There were six studies that measured endothelial derived EV using various endothelial specific markers; CD31+ (Li and Qin 2015; Jung et al. 2009; Schrick et al. 2022), (CD144+) (Li and Qin 2015), CD105+ (Simak et al. 2006) and CD146+ (Chiva‐Blanch et al. 2016; Agouni et al. 2019) EVs were found increased in IS when compared with HC (*p* < 0.05 for all). A further study confirmed increased endothelial EV at stroke onset (Agouni et al. 2019) compared to HC and the number of endothelial EV remained significantly increased at 5‐ and 30‐days post‐incident. Interestingly, two studies reported endothelial EV concentrations were the same in IS, HC and stroke mimic groups (*p* > 0.05) (Eyileten et al. 2022; Williams et al. 2007); however, the latter may be limited due to analysing a very small IS sample group of 10 patients.

Only one study determined endothelial EV number in TIA patients. The study found endothelial EVs (CD146+) were significantly higher at sample acquisition compared to HC, similar to that seen in IS where endothelial EV number remained increased when remeasured at 5‐ and 30‐days post‐TIA (Agouni et al. 2019).

#### 3.5.3 Erythrocytes

Red blood cell EVs were typically characterised using CD235a as a marker. Two studies showed significantly elevated CD235A+ (Chiva‐Blanch et al. 2016; Agouni et al. 2019) EV at onset of IS compared with HC (*p* < 0.01 for both). One study also measured at 5‐ and 30‐days post‐stroke and found the level of erythrocyte derived EVs had returned to normal with no significant difference between IS and HC groups. One contradicting study found no difference in CD235a+ EV between IS and HC groups (Simak et al. 2006). When comparing the studies, samples were collected within 48 h of stroke onset for all patients across the three studies.

Moreover, in one study that determined red blood cell EV in TIA patients, CD235A+ EV was significantly increased in TIA patients at onset compared to HC and levels returned to normal at 5‐ and 30‐days post‐incident (Agouni et al. 2019).

#### 3.5.4 White Blood Cells

White blood cell EVs were characterised using several standard markers, dependent on the cell type the study focussed on. Four studies used CD45+ as a marker for leukocyte‐derived EV (Chiva‐Blanch et al. 2016; Eyileten et al. 2022; He et al. 2017; Agouni et al. 2019) and found the population to be increased in IS compared with HC (*p* < 0.01, *p* = 0.005, *p* = 0.027 and *p* < 0.01, respectively). Lymphocyte‐derived EVs with surface markers CD3+ (Chiva‐Blanch et al. 2016) and CD4+ (He et al. 2017) were found elevated in IS compared with HC (*p* < 0.01 and *p* = 0.038). Monocyte‐derived EVs with surface markers CD11A+, CD142+ (Chiva‐Blanch et al. 2016) and CD14+ (He et al. 2017) were significantly increased in IS groups compared to HC (*p* < 0.01 and *p* = 0.013). Granulocyte‐derived EVs with surface markers CD15+ (He et al. 2017) and CD66B+ (Agouni et al. 2019) were significantly higher in IS than HC (*p* = 0.021 and *p* < 0.01). All types of white blood cell‐derived EV that were assessed across four studies showed complimentary increases in IS patients.

In agreement with results from IS patients, one study identified elevated leukocyte (CD45+) and granulocyte (CD66B+) derived EV in patients who experienced a TIA compared to HC (*p* < 0.01) (Agouni et al. 2019).

#### 3.5.5 Brain Cells

Four studies investigated the production of EV from brain cells in plasma of IS patients, of which two were specifically focussed on EVs produced from astrocytes (Edwardson et al. 2024; Forró et al. 2024).

Both studies reported significantly elevated levels of astrocyte derived EVs up to one month post‐IS when compared to HCs (*p* > 0.05). This poses an important question of whether they hold potential as biomarkers of blood–brain barrier breakdown due to associations with lesion size being reported in one study (Edwardson et al. 2024), and that this difference may reflect dynamic changes that occur from a cellular level during the first month post‐stroke.

One study assessed pericyte‐derived EV, using CD140b+, which was significantly elevated in IS compared to HC at 12–14 h (*p* = 0.0041) after stroke onset and remained elevated for up to 6 days (*p* = 0.0237) (Gaceb et al. 2024). In addition, EV derived from neural progenitor cells (CD34+, CD56+) were also significantly higher in IS patients (*p* < 0.001) at stroke onset (Chiva‐Blanch et al. 2016). At the time of this review, no literature on brain cell‐derived EV has been published regarding TIA patients.

#### 3.5.6 Mesenchymal Stem Cells

One study investigated EV production from mesenchymal stem cell (MSC) using a variety of surface marker expression (CD105+, CD90+, CD73+ and CD144−) and were significantly higher in IS compared to HC (Kim et al. 2012). It is of note that the lack of CD144 expression on the EV surface deems the EV not of endothelial origin, this is important as CD105 is also highly expressed on the surface of endothelial EV due to its angiogenic role. No studies at this timepoint had reported on MSC EV in TIA patients.

### 3.6 EV Associated Clinical Factors Severity

EC EVs (Simak et al. 2006) (*r* = 0.520, *p* = 0.002), astrocyte‐derived EVs (Edwardson et al. 2024) (*p* = 0.04) and MSC‐derived EVs (Kim et al. 2012) (*r* = 0.263, *p* = 0.005) levels were positively correlated with brain lesion volume in IS patients. A positive association was found between stroke severity and EC EV in two studies (Li and Qin 2015; Simak et al. 2006). One study showed endothelial CD105+ EVs were correlated with stroke severity (Simak et al. 2006). Li and Qin (2015) showed that EC CD144+ EVs were correlated with stroke severity (*r* = 0.750, *p* = 0.020). According to another study, monocyte derived EVs (CD14+) were significantly correlated with stroke severity (He et al. 2017) (*r* = 0.355, *p* = 0.019).

### 3.7 EV Functional Characteristics

#### 3.7.1 Activation Markers

EVs have the potential to influence downstream function due to their potential in facilitating cell‐to‐cell communication. When cells become activated, they produce EV with activation markers on their surface that can influence other cell types. A marker of platelet activation, CD62P+, was reported as significantly higher in IS patients in comparison with HC in three studies (Rosińska et al. 2019; Chiva‐Blanch et al. 2016; Agouni et al. 2019) (*p* = 0.008, *p* < 0.001 and *p* < 0.01, respectively). Similarly, Burrello et al. (2021) found elevated CD62P+ EVs in TIA patients (*p* < 0.05). In agreement to this, one study showed that CD62P+ EVs were elevated at TIA onset and remained elevated for up to 30 days post‐incident (Agouni et al. 2019) (*p* < 0.05). The increase in activated platelet EV is taken to represent the apparent increased platelet activation present in both IS and TIA patients.

Five studies focussed on endothelial EV populations. The concentration of activated (CD62E+) endothelial EV was considerably higher in IS patients than HC (Chiva‐Blanch et al. 2016; Li and Qin 2015; Agouni et al. 2019) (*p* < 0.001, *p* < 0.05, *p* < 0.01) and risk factor (RF) (Jung et al. 2009) (*p* < 0.001) groups. However, one study with a small IS population size, reported no difference in CD62E+ EVs when compared to stroke mimics (Williams et al. 2007) (*p* = 0.579). Patients who presented with similar symptoms but were not diagnosed with a stroke were classed as ‘stroke mimics’, the similarity between groups may reflect the extent of endothelial activation present in both populations. However, no data from HCs were included in this study, which limits our ability to associate this finding specifically to these patient characteristics. In regard to endothelial activation in TIA patients, one study reported elevated CD62E+ EVs post‐TIA onset, with continued elevation present at 30 days post‐incident (Agouni et al. 2019) (*p* < 0.05).

#### 3.7.2 Coagulation

Following a stroke, the apparent imbalance of coagulation factors observed may be reflected in the EV profiles of IS and TIA patients. EVs with procoagulant markers were increased in IS and TIA patients compared to HCs. Tissue factor (TF), a high‐affinity receptor and cofactor for factor VII/VIIa, is a key factor in the coagulation cascade. TF+ positive EVs were higher in IS (Świtońska et al. 2015; Lundström et al. 2020) (*p* < 0.001, *p* < 0.0001) and TIA (Burrello et al. 2021) (*p* < 0.05) compared to HC. A study by Słomka et al. (2017) found no difference in TF+ EVs between low and high CRP IS patients, indicating no association between these two factors (*p* > 0.05). However, no control group was presented to specifically ascribe elevated TF+ EV levels to these patient groups. Phosphatidylserine (PS) is also capable of promoting coagulation and the levels of PS+ EVs was significantly higher in IS groups compared to HCs in multiple studies (Yao et al. 2017; Simak et al. 2006; Lundström et al. 2020). In two of the three studies, the elevated PS+EVs were of platelet origin (Yao et al. 2017; Lundström et al. 2020) (*p* < 0.05 for both) and in the other were of endothelial origin (Simak et al. 2006) (*p* = 0.002). Proteins involved in the coagulation cascade (such as fibrinogen alpha‐chain, fibrinogen beta chain) were found to be upregulated in EVs from IS patients compared with HC (Datta et al. 2014). This study also identified upregulation of focal adhesion proteins, such as Integrin Alpha 11b, talin‐1 and filamin A. Adhesion molecule markers were also elevated on EVs from TIA patients, with significantly higher CD44, CD326 and CD2 positivity compared to HCs (Burrello et al. 2021) (*p* < 0.05 for all).

#### 3.7.3 Neuronal Proteins

Proteins involved in the maintenance and development of neuronal cells, STX‐1 and SNAP‐25, were significantly increased in neuronal‐derived EVs from IS patients compared to HC (Cappelletti et al. 2022) (*p* < 0.0001). One study identified a multitude of proteomic changes in pericyte‐derived EVs dependent on time from IS insult (Gaceb et al. 2024). At 0–6 h post‐IS, SIRT2, CD40, FGF21, GNDF, CD244, MCP‐4, IL17, IL‐5, IL‐6 and IL‐2 protein levels were decreased, marking a reduced production of inflammatory‐related molecules. However, over the course of 24 h to 6 days, there is a parallel increased production of pro‐angiogenic and inflammatory proteins, specifically CD40, MMP‐1, VEGFa and CXCL5 (*p* < 0.05 for all). Kowalski et al. (2023) also reported on the activation of the inflammatory cascade, establishing that it is mimicked in the profile of EVs post‐IS, with MMP‐9, CXCL‐4, CRP, IL‐6 and OPN concentrations being significantly higher than HCs (*p* < 0.01).

A study by Forró et al. (2024) identified an increase in neuroprotective protein expression, specifically AQP4 and GDNF, in EVs isolated from stroke patients at 24‐h post‐insult in comparison to HCs (*p* < 0.05).

### 3.8 Studies in Accordance With the MISEV Guidelines

Broadly, the MISEV guidelines can be split into four components: EV nomenclature, EV source, EV isolation and EV characterisation. Surprisingly, only one paper out of the 31 analysed referred to the MISEV guidelines that were intended to standardise EV data across research. The literature that was analysed in this systematic review showed each paper used one exclusive term to describe EV populations (listed in Table 2). However, five studies were published pre‐publication of the MISEV2014 guidelines, and all used the term microparticles (Kim et al. 2012; Simak et al. 2006; Jung et al. 2009; Williams et al. 2007) or microvesicles (Datta et al. 2014). It should be noted that most recent publications (from 2020 onwards) have been utilising the recommended term ‘extracellular vesicle’.

**TABLE 2:** Nomenclature term used in the studies of EV and TIA or ischaemic stroke.

Nomenclature term	Number of studies	Reference
Extracellular vesicle (EV)	13	Kowalski et al. 2023; Cappelletti et al. 2022; Otero‐Ortega et al. 2021; Eyileten et al. 2022; Burrello et al. 2021; Agouni et al. 2019; Picciolini et al. 2023; Edwardson et al. 2024; Forró et al. 2024; Maciejewska‐Renkowska et al. 2024; Forró et al. 2024; Escudero‐Guevara et al. 2025; Reymond et al. 2025
Exosomes	1	Lu et al. 2023
Microvesicles (MVs)	4	Datta et al. 2014; Gaceb et al. 2024; Rosińska et al. 2019; Lundström et al. 2020
Microparticles (MPs)	13	Yao et al. 2017; Chiva‐Blanch et al. 2016; Simak et al. 2006; Chen et al. 2015; Jung et al. 2009; Świtońska et al. 2015; Pawelczyk et al. 2017; He et al. 2017; Li and Qin 2015; Kim et al. 2012; Williams et al. 2007; Słomka et al. 2017; Schrick et al. 2022

Regarding MISEV category two, all sources of EV analysed in these studies were of blood origin (plasma, serum). MISEV category three; isolation methods used in studies varied considerably and details can be found in Table S1, with only a few studies meeting the recommended guidelines to maximise EV recovery and purity. The fourth MISEV domain is EV characterisation. The literature has become a rich source of information relating to EV content and character that has excelled in recent years. Most papers used flow cytometry to characterise both EV‐specific and cell origin markers, but other techniques included ELISAs, western blotting and Nanosight tracking analysis.

## 4 Brief Discussion

This systematic review included 31 studies investigating EV in IS and TIA. Investigating the role of EVs in these conditions offers valuable insights into the underlying pathophysiology and presents an opportunity for identifying potential biomarkers of disease progression and recurrent risk. Despite considerable variability in EV isolation and characterisation methodologies within the included studies, one consistent finding was the significant involvement of EV in both IS and TIA.

Findings on total EV concentration were inconsistent across studies. Over 60% of IS studies reported no significant difference in total EV concentration between IS patients and controls, a trend mirrored by the limited studies in TIA patients. Data on EV size were even more limited. One IS study reported no difference in mean EV size between IS patients and controls, while a single TIA study found significantly smaller EVs compared to controls. These conflicting findings likely stem from methodological differences in EV isolation, as well as small sample sizes in several studies. Standardised measurement of EV size should become routine in population‐based EV research, as many downstream analytical techniques, such as flow cytometry, rely on accurate size‐based discrimination to confirm EV identity, but also have intrinsic limitations.

Although the picture regarding total EV population is inconclusive, multiple studies reported elevated levels of EVs released from certain cell types, including ECs, platelets and leukocytes, in both IS and TIA patients. These EV sub‐populations were implicated in multiple pathways relevant to stroke pathophysiology, including coagulation, inflammation and angiogenesis. Numerous studies identified higher platelet‐derived EVs, carrying activation markers in IS patients, suggesting a role in amplifying thrombotic responses. In longitudinal studies, both platelet and activated platelet EV remained elevated up to 30‐days post‐event in both IS and TIA, indicating a prolonged prothrombotic state. In agreement, several studies found increased levels of EVs with procoagulant surface markers (e.g., TF+, PS+) in both patient populations, further supporting the hypothesis that EV may contribute to recurrent stroke risk, even in patients with transient events.

Elevated levels of endothelial EV were reported in 24% of studies where TIA and IS were compared to controls. Markers of endothelial activation, such as CD62E+, were significantly upregulated post‐ischemic insult, suggesting hypoxia‐induced endothelial dysfunction contributes to EV release (Burnley‐Hall et al. 2017). In one study, sustained cellular activation was observed on EVs for up to 30 days post‐event, highlighting the potential of EV to serve as long‐term indicators of vascular injury. Additionally, surface markers linked to numerous biological processes, such as cell adhesion, inflammation, platelet function, neuroinflammation, angiogenesis and vascular remodelling, were more abundant in EV from IS patients compared with controls.

This review identified several promising avenues for EV as prognostic biomarkers, specifically those of endothelial origin. In two studies, endothelial EV levels correlated positively with clinical markers of stroke severity and brain lesion volume, suggesting their potential utility in assessing ischaemic burden. However, no studies reported similar associations for TIA patients, highlighting an important gap in current research that warrants further investigation.

### 4.1 Limitations

The methodological appraisal score used in this review was developed by the authors for descriptive purposes and has not been formally validated. The score is only intended to aid interpretability and transparency when comparing heterogeneous EV studies, rather than to generate definitive quality rankings. In addition, interpretation should be considered in the context of the publication year. Five of the included studies predated the introduction of the MISEV guidelines and thus could not explicitly reference them. The search terms used within this review were intentionally focused on TIA and IS. This may have led to the exclusion of studies with mixed stroke populations or that focus specifically on haemorrhagic stroke.

A major limitation across the reviewed studies was the heterogeneity in EV nomenclature, isolation protocols and characterisation methods. This underscores the urgent need for standardisation in EV research. Although the MISEV guidelines (Welsh et al. 2024) provide a comprehensive framework for standardisation, only one of the 20 studies published after 2014 cited these guidelines, pointing to a significant lack of adherence. Despite this, the body of research reviewed here reflects substantial progress in understanding the diagnostic and prognostic potential of EVs in cerebrovascular disease.

A key limitation that is present across the studies presented in this review relates to detection methods that rely on flow cytometry and other marker‐based approaches. Conventional flow cytometry has limited sensitivity for small vesicle populations, with thresholds approaching the size range in which many EV populations sit. This is exacerbated by results being heavily reliant on machine configuration, gating strategies and calibration. For other marker‐based enumeration, it is imperative to consider antibody specificity and non‐specific binding that could lead to signal contamination. Variability in the reporting of analytical parameters further limits the cross‐comparability of the studies presented, further supporting the need for standardised reporting and characterisation methodologies when investigating EV (Royo et al. 2020).

Overall, the limited evidence suggests that TIA patients exhibit EV profiles similar to those of IS patients, supporting the notion that EVs could serve as biomarkers for identifying individuals at risk of stroke recurrence, even in the absence of permanent neurological damage. Although the qualitative importance of EVs in IS and TIA is evident, greater consistency in methodological standards is essential to enable meaningful comparisons and translational applicability.

## 5 Conclusion

EV hold promise for advancing our understanding of IS pathology and have potential as diagnostic and prognostic biomarkers. However, the current literature is heterogeneous, with inconsistent findings for total EV concentration, suggesting that specific EV populations or indeed their protein expression profiles may be more informative than general EV counts. Future studies investigating EVs in TIA and IS should adopt standardised methodologies, such as those outlined in MISEV, to ensure consistency, enhance reproducibility and facilitate clinical translation of research findings.

## Ethical Statement

This study contains no new data, however, received ethical approval under Cardiff Metropolitan University Ethics Framework (PGT‐9454).

## Conflicts of Interest

The authors declare no conflicts of interest.

## Supporting information



Supporting Information: jex270124‐sup‐0001‐SuppMat.docx

## Data Availability

This article reports a systematic review of previously published studies and does not include any new primary data. All data supporting the findings are contained within the referenced publications.
