# Supplementary material for: The Role of Extracellular Vesicles in Transient Ischaemic Attacks and Ischaemic Stroke: A Systematic Review
Source: J Extracell Biol. 2026 Mar 13;5(3):e70124. doi: 10.1002/jex2.70124 (PMC13097698; doi:10.1002/jex2.70124)
Supplement: Supplementary file 1 — Supporting Information: jex270124‐sup‐0001‐SuppMat.docx [file JEX2-5-e70124-s001.docx]

Supplementary Materials

s1. Search Syntax

Key search terms:

Transient Ischaemic attack(s) / Transient Ischemic attack(s)

Ischaemic Stroke(s) / Ischemic Stroke(s)

Extracellular vesicle(s)

Exosome(s)

Microvesicle(s)

Microparticle(s)

PubMed

(“transient ischaemic attack” OR “ischaemic stroke” OR “transient ischemic attack” OR “Ischemic stroke”) AND (“extracellular vesicl*” OR exosom* OR microvesicl* OR microparticle*) NOT review[publication type] AND English[language]

Scopus

((TITLE-ABS-KEY (“transient ischeamic attac*”)) OR (TITLE-ABS-KEY (“transient ischemic attac*”)) OR (TITLE-ABS-KEY (“ischeamic strok*”)) OR (TITLE-ABS-KEY (“ischemic strok**”)) AND (TITLE-ABS-KEY (“extracellular vesicl*”)) OR (TITLE-ABS-KEY (“exosom*”)) OR (TITLE-ABS-KEY (“Microvesicl*”)) OR (TITLE-ABS-KEY (“microparticl*”))) AND NOT (review)

Web of Science

((transient ischeamic attac* OR transient ischemic attac* OR ischeamic strok* OR ischemic strok*) AND (extracellular vesicl* OR exosom* OR microvesicl* OR microparticle*))

(and filtered for NOT review)

s2.Table

| **Allocated number** | **Reference** | **Study populations (n/5)** | | **TIA/stroke Diagnosis**  **(n/2)** | **EV isolation (n/3)** | **EV characterisation (n/3)** | **Total (n/13)** | **Score** |
| --- | --- | --- | --- | --- | --- | --- | --- | --- |
|  |  | **Sample size (n/3)** | **Control groups (n/2)** |  |  |  |  |  |
| 1 (23) | 10.1161/STROKEAHA.122.041422 | 1  Small sample size of both groups  8 confirmed Stroke  7 stroke mimic | 1 | 2  MRI or CT | 2 | 1  No EV concentration or size  Limited characterisation | 7 | **0.54** |
| **2 (17)** | 10.1038/s41598-022-18719-2 | 3  30 ischaemic stroke | 2 | 2  MRI or CT | 2 | 1  No EV concentration or size  Surface proteins | 9 | **0.69** |
| **3 (14)** | 10.3390/biomedicines9070786 | 3  55 cortical sub stroke  26 subcortical stroke  22 control | 1 | 2  MRI or CT | 2  3000xg for 15 mins  ExoQuick Ultra EV precipitation solution  SEC for brain derived EV | 3  Size and concentration by NTA  WB to confirm EV proteins/ELISA  Proteomics and confirmatory WB | 11 | **0.84** |
| 4 (15) | 10.1007/s12975-016-0501-7 | 3  79 AIS  35 Risk factor matched  32 healthy | 2 | 2 CT or MRI | 2  200xg 13 min  1500xg for 20 min  13000xg for 2 min  20000xg for 45min | 1  Flow for cell surface proteins | 10 | **0.76** |
| **5 (16)** | 10.1371/journal.pone.0094663 | 3  LACI 45  Healthy 17 | 1 | 2  “brain imaging” not descriptive of which | 2  4000xg for 30 min  4000xg for 30 min  12000xg for 30 min  Ultra 30000xg for 2h  Ultra 200000xg for 2h15 | 2  Proteomics  Western Blot  Mass Spc | 10 | **0.76** |
| **6 (31)** | 10.1161/strokeaha.123.045720 | 3  39+39 control | 1 | 2  CT | 1  ISEV not mentioned; differential centrifugation   - 750*g* for 20 minutes - 1500*g* for 20 minutes   21 000*g*, 45 minutes | 2  No NTA   - Flow used for characterisation of proteins on surface - Protein Content also determined | 8 | **0.61** |
| **7 (18)** | 10.1016/j.thromres.2019.01.014 | 3  40 IS after large artery atherosclerosis  + 32 IS after small  + 67 control | 1 | 2  CT or MRI | 1  ISEV not mentioned; differential centrifugation   - 1500 ×  *g* for 20 min - 13000 ×  *g* for 2 min   30 min at 18890 ×  *g* | 2   - No NTA but Number determined by flow, - 7 protein markers by flow | 9 | **0.69** |
| **8 (19)** | 10.1371/journal.pone.0148176 | 3  44 patients + 44 control | 1 | 2  CT or MRI | 1  ISEV not mentioned; differential centrifugation   - 250xg, 15min - 11000×g, 10min, RT - 11000xg, 3min, R   20000×g for 30min. | 2  No NTA,   - EV concentration by flow - Surface protein markers by flow   0 | 9 | **0.69** |
| **9 (20)** | 10.3390/ijms23094530 | 3  28 + 35 control | 1 | 2  CT or MRI | ISEV not mentioned  Differential centrifugation did not meet criteria;   - double centrifugation as previously described   reported results according to the standardized framework (MIFlowCyt-EV) | 2  Concentration by Flow  Cell surface markers by flow  miRNA was additionally analysed | 9 | **0.69** |
| **10 (32)** | 10.1111/j.1538-7836.2006.01911.x | 3  41 + 23 controls | 1 | 2 CT or MRI | 1  ISEV not stated differential centrifugation remit   - 15 min at 2700 × ***g*** at 10 °C. - 5 min at 2700 × ***g***   19 800 × ***g*** at 10 °C | 1  No NTA,   - 6 protein markers by flow | 8 | **0.61** |
| **11 (33)** | 10.1016/j.jstrokecerebrovasdis.2015.06.018 | 3  112+35 controls | 1 | 2  MRI | 1  ISEV not mentioned; differential centrifugation   - 800  *g* for 30 minutes - 1500  *g* for 15 minutes   35,000  *g* for 15 minutes | 1  No NTA   - analysis of surface proteins by flow cytometry | 8 | **0.61** |
| **12 (34)** | 10.1002/ana.21681 | 3  73 + 275 control | 1 | 2  Symptoms + diffusion-weighted imaging | 1  ISEV not mentioned; differential centrifugation   - 1,500*g* for 15 minutes - 2 minutes at 13,000*g*   3,000*g* for 15 minutes | 1  No NTA   - Thorough analysis of cell surface proteins by flow cytometry | 8 | **0.61** |
| **13 (35)** | 10.1111/ene.12591 | 3  73 ischaemic stroke (split into thrombolysed or not)  30 controls | 1 | 2  CT | 1  1500xg 15 min  13000xg for 2 min  1500xg for 20 mins | 1  Protein levels by ELISA | 8 | **0.61** |
| **14 (36)** | 10.1161/STROKEAHA.120.033170 | 3  20 TIA  20 Control | 1 | 2  MRI | 0  Isn’t clear, just says bloods were centrifuged. | 2  Size and concentration by NTA  Bead based EV capture FLOW  37 surface antigens tested | 8 | **0.61** |
| **15 (37)** | 10.1007/s12975-019-00777-w | 3  211 IS/TIA  53 Control | 1 | 1  NIHSS stroke score | 2-differential centrifugation  2000xg for 20 min  2000xg for 20 min  13000xg for 2 min | 1  Flow for surface markers | 8 | **0.61** |
| **16 (24)** | 10.5114/aoms.2017.65816 | 3   1. experimental groups   (total 73)  1 control group 21 | 1 | 2 CT | 0 | 1 size and one protein marker by flow | 7 | **0.54** |
| **17 (25)** | 10.1016/j.thromres.2017.03.025 | 3  76 IS  +76 control | 1 | 2 CT or MRI | 0  ISEV not mentioned and differential centrifugation not met  1700xg for 10 min  3000xg for 30 min | 1  ELISA for cell surface  FLOW for cell surface | 7 | **0.54** |
| **18 (26)** | 10.1016/j.thromres.2014.12.006 | 3  68 stroke  61 controls | 1 | 2 – CT and MRI | 0 ISEV not mentioned differential centrifugation not met.  1500xg for 10 min  2700xg for 30 min | Cell surface expression of proteins by flow | 7 | **0.54** |
| **19 (27)** | 10.1186/s12883-023-03348-7 | 3  75 + 75 control | 1 | 2  Symptoms and MRI | 0  ISEV not mentioned; used  Spinning not satisfied;   - 2000×g for 30 min - exosome isolation reagent   10,000 ×g for 10 min | 1  No NTA or flow   - ELISA for one surface protein   And Western Blot for surface CD63 and CD81 | 7 | **0.54** |
| **20 (28)** | 10.1371/journal.pone.0037036 | 3  111+50 control | 1 | 2  MRI | 0  ISEV not mentioned; only 2 differential centrifugation steps   - 1800 g for 15 minutes   10 min at 19,800 g | 1  No NTA   - Thorough analysis of proteins by flow cytometry; 9 surface proteins   Confirmed in vitro | 7 | **0.54** |
| **21 (38)** | 10.1197/j.aem.2007.04.009 | 1  10 AIS  10 Mimic | 1 | 2  CT | 0 ISEV not mentioned, differential centrifugation not met  180xg for 10 min  20,000xg for 20 min | 2  Number and size by flow  Cell surface proteins by flow | 6 | **0.46** |
| **22 (42)** | 10.1177/0004563216663775 | 2  66 stroke | 0 | 2 CT | 0  ISEV not mentioned and differential centrifugation not met  1500xg for 15min  13000xg for 2 min | 1  ELISA for cell surface  ZYMUPHEN MP activity kit for concentration and procoagulant activity | 5 | **0.38** |
| **23 (39)** | 10.3389/fneur.2019.00251 | 3  Control 24  Acute Ischaemic Stroke 66  TIA 21 | 1 | 0 | 0  ISEV not mentioned, differential centrifugation does not meet criteria  270xg 20min  1500xg for 15 min | 2  Flow for surface markers and fluro beads for EV count | 6 | **0.46** |
| **24 (29)** | 10.3390/ijms24097937 | 1  19 + 20 controls | 1 | 0 – just states diagnosis of ischaemic stroke | 2  SEC  And ultracentrifugation for different portions | 3  Size and Concentration by NTA  Cell surface and internal protein expression | 7 | **0.54** |
| **25 (43)** | 10.31083/j.fbl2705158 | 1  18 patients  But has a control group of 20 | 1 | 0 | 1  ISEV not mentioned,  differential spinning, 2500 × g for 20 minutes   - 2500 × g for further 20 minutes   18000 × g for 10 minutes | 1  No NTA,   - 6 surface protein markers by flow | 4 | **0.31** |
| **26 (30)** | 10.1038/s41598-024-55983-w | 3  Ischaemic stroke N=58  Control  N=46 | 1 | 2  All participants MRI – diagnosis | 0  ISEV not mentioned  Elisa sandwich assay – labelling for CD9 capture and then markers of interest | 1  No flow or NTA  Measured using ELISA assay quantification for brain cell origin – ADE/NDE or ODE | 7 | **0.54** |
| **27 (40)** | 10.3390/ijms25115726 | 1  AIS  N=18  Control  Healthy  N=9 | 1 | 2  CT scan mentioned | 1  3000xg for 15 mins  ExoQuick Ultra EV precipitation solution  ADEVs isolated using beads with GLAST marker and eluted off. | 1  EVs were captured on (EV+ marker beads; CD9.CD81,CD63) and showed enrichment of EV by Flow cytometry – no concentration or size stated.  Western blot of EV to detect GFAP. | 6 | **0.46** |
| **28 (21)** | 10.3390/ijms252011219 | 3  IS  N=168  Disease matched controls  N=63  HC  N=21 | 2 | 2  CT AND/OR MRI diagnosis | 1  Differential centrifugation | 1  Flow cytometry for platelet derived EVs | 9 | **0.69** |
| **29 (41)** | 10.3390/ijms252212471 | 1  AIS = 18  HC = 9 | 1 | 2  CT diagnosis | 1  3000xg for 15 mins  ExoQuick Ultra EV precipitation solution  ADEVs isolated using beads with GLAST marker and eluted off. | 1  Western blot for EV cargo | 6 | **0.46** |
| **30 (10)** | 10.1113/jp285907 | 1  IS  Mild = 9  Severe = 9  HC  N=9 | 1 | 1  Symptoms  No mention of CT or MRI | 2  Differential centrifugation and ultracentrifugation | 2  EV size and concentration by NTA  EV content by western blot | 7 | **0.54** |
| **31 (22)** | 10.1016/j.jprot.2025.105468 | 2  Stroke patients  N=50  Subgroups:  10 HS  10 IS with LVO  10 IS wo LVO  10 TIA  10 stroke mimics  HC  N=10 | 1 | 2  Neuroimaging mentioned in study protocol | 2  EV isolated by SEC | 2  NTA – EV size and concentration  No ‘surface marker’ method used  Western blot and Mass spectrometry for protein analysis | 9 | **0.69** |
